# MetDecode: methylation-based deconvolution of cell-free DNA for noninvasive multi-cancer typing

**DOI:** 10.1093/bioinformatics/btae522

**Published:** 2024-08-23

**Authors:** Antoine Passemiers, Stefania Tuveri, Dhanya Sudhakaran, Tatjana Jatsenko, Tina Laga, Kevin Punie, Sigrid Hatse, Sabine Tejpar, An Coosemans, Els Van Nieuwenhuysen, Dirk Timmerman, Giuseppe Floris, Anne-Sophie Van Rompuy, Xavier Sagaert, Antonia Testa, Daniela Ficherova, Daniele Raimondi, Frederic Amant, Liesbeth Lenaerts, Yves Moreau, Joris R Vermeesch

**Affiliations:** Dynamical Systems, Signal Processing and Data Analytics (STADIUS), Department of Electrical Engineering, KU Leuven, Leuven, 3001, Belgium; Laboratory for Cytogenetics and Genome Research, Department of Human Genetics, KU Leuven, Leuven, 3000, Belgium; Laboratory for Cytogenetics and Genome Research, Department of Human Genetics, KU Leuven, Leuven, 3000, Belgium; Laboratory for Cytogenetics and Genome Research, Department of Human Genetics, KU Leuven, Leuven, 3000, Belgium; Gynaecological Oncology, Department of Oncology, KU Leuven, Leuven, 3000, Belgium; Gynaecology and Obstetrics, University Hospitals KU Leuven, Leuven, 3000, Belgium; Multidisciplinary Breast Centre, University Hospitals Leuven, Leuven, 3000, Belgium; Laboratory of Experimental Oncology, Department of General Medical Oncology, University Hospitals Leuven, KU Leuven, Leuven, 3000, Belgium; Department of Oncology, GZA Ziekenhuis, Antwerp, 2610, Belgium; Laboratory of Experimental Oncology, Department of General Medical Oncology, University Hospitals Leuven, KU Leuven, Leuven, 3000, Belgium; Digestive Oncology Unit, University Hospital Gasthuisberg, Leuven, 3000, Belgium; Laboratory of Tumour Immunology and Immunotherapy, Department of Oncology, Leuven Cancer Institute, KU Leuven, Leuven, 3000, Belgium; Gynaecological Oncology, Department of Oncology, KU Leuven, Leuven, 3000, Belgium; Gynaecology and Obstetrics, University Hospitals KU Leuven, Leuven, 3000, Belgium; Gynaecology and Obstetrics, University Hospitals KU Leuven, Leuven, 3000, Belgium; Translational Cell & Tissue Research, Department of Pathology, KU Leuven, Leuven, 3000, Belgium; Translational Cell & Tissue Research, Department of Pathology, KU Leuven, Leuven, 3000, Belgium; Translational Cell & Tissue Research, Department of Pathology, KU Leuven, Leuven, 3000, Belgium; Department of Woman, Child and Public Health, Fondazione Policlinico Universitario Agostino Gemelli, IRCCS, Rome, 00168, Italy; Obstetrics and Gynaecology, First Faculty of Medicine, Charles University and General University Hospital in Prague, Prague, Czech Republic; Dynamical Systems, Signal Processing and Data Analytics (STADIUS), Department of Electrical Engineering, KU Leuven, Leuven, 3001, Belgium; Gynaecological Oncology, Department of Oncology, KU Leuven, Leuven, 3000, Belgium; Gynaecology and Obstetrics, University Hospitals KU Leuven, Leuven, 3000, Belgium; Department of Gynaecologic Oncology, Netherlands Cancer Institute, Amsterdam, 1066 CX, The Netherlands; Gynaecological Oncology, Department of Oncology, KU Leuven, Leuven, 3000, Belgium; Dynamical Systems, Signal Processing and Data Analytics (STADIUS), Department of Electrical Engineering, KU Leuven, Leuven, 3001, Belgium; Laboratory for Cytogenetics and Genome Research, Department of Human Genetics, KU Leuven, Leuven, 3000, Belgium

## Abstract

**Motivation:**

Circulating-cell free DNA (cfDNA) is widely explored as a noninvasive biomarker for cancer screening and diagnosis. The ability to decode the cells of origin in cfDNA would provide biological insights into pathophysiological mechanisms, aiding in cancer characterization and directing clinical management and follow-up.

**Results:**

We developed a DNA methylation signature-based deconvolution algorithm, MetDecode, for cancer tissue origin identification. We built a reference atlas exploiting *de novo* and published whole-genome methylation sequencing data for colorectal, breast, ovarian, and cervical cancer, and blood-cell-derived entities. MetDecode models the contributors absent in the atlas with methylation patterns learnt on-the-fly from the input cfDNA methylation profiles. In addition, our model accounts for the coverage of each marker region to alleviate potential sources of noise. *In-silico* experiments showed a limit of detection down to 2.88% of tumor tissue contribution in cfDNA. MetDecode produced Pearson correlation coefficients above 0.95 and outperformed other methods in simulations (*P* < 0.001; *T*-test; one-sided). In plasma cfDNA profiles from cancer patients, MetDecode assigned the correct tissue-of-origin in 84.2% of cases. In conclusion, MetDecode can unravel alterations in the cfDNA pool components by accurately estimating the contribution of multiple tissues, while supplied with an imperfect reference atlas.

**Availability and implementation:**

MetDecode is available at https://github.com/JorisVermeeschLab/MetDecode.

## 1 Introduction

Cell-free DNA (cfDNA) contains the genomic and epigenetic signatures of the cells of origin (Aucamp *et al.* 2018). Circulating-tumor DNA (ctDNA), defined as the cfDNA fraction derived from tumor cells (Schwarzenbach *et al.* 2011), is now widely explored as a noninvasive biomarker for cancer screening and diagnosis (Chen *et al.* 2020).

CfDNA-based screening exploiting cancer-specific single nucleotide mutations and copy number alterations (CNAs) can often detect the presence of abnormal signals indicative of cancer but cannot ascertain the tumor’s origin or the cancer type (Chan *et al.* 2013, Lenaerts *et al.* 2019, Meng *et al.* 2021, Boons *et al.* 2022, Gao *et al.* 2022, Song *et al.* 2022). Especially for metastatic disease of unknown primary, profiles do not readily allow the identification of the tissue of origin (TOO) albeit this would be of clinical value (Qaseem *et al.* 2019). When performing noninvasive prenatal screening for fetal chromosomal aneuploidies, incidental occult maternal malignancies can be detected without insights into the origin (Bianchi *et al.* 2015, Lenaerts *et al.* 2020, Rengifo *et al.* 2020). If the TOO or cancer type could be deduced from cfDNA analysis, this would tremendously speed up the diagnosis and start of treatment, hence streamlining subsequent clinical follow-up, reducing costs and minimizing the need for extensive radiologic imaging (Etzioni *et al.* 2003). This might reduce the anxiety associated with a positive screening test outcome for patients.

Methods to deduce the origin of cfDNA fragments have been based on epigenetic markers such as nucleosome positioning, fragmentation, and methylation profiles (Sun *et al.* 2015, Lehmann-Werman *et al.* 2016, Snyder *et al.* 2016, Stanley *et al.* 2024). In fact, these features change according to the gene expression programs of a specific cell type (Dor and Cedar 2018), offering the possibility to identify the different components of the cfDNA pool, alongside an estimation of the relative proportion of each of them. Tumor-associated methylation modifications have been described during cancer initiation and progression (McMahon *et al.* 2017). Hence, they represent a promising marker for early cancer TOO identification.

Recently, several algorithms have been developed to deconvolute the plasma cfDNA composition based on methylation profiles. Typically, reference atlases consisting of either normal tissues or cell-type-specific methylation markers are used to identify tissue-specific methylation signals (Kang *et al.* 2017, Lutsik *et al.* 2017, Li *et al.* 2018, Moss *et al.* 2018, Arneson *et al.* 2020, Erger *et al.* 2020, Caggiano *et al.* 2021, Keukeleire *et al.* 2022, Zhang *et al.* 2022). MethAtlas (Moss *et al.* 2018) and cfNOMe (Erger *et al.* 2020), built on nonnegative least squares (NNLS) and constrained programming, respectively, model cfDNA mixtures as exact linear combinations of the reference cell types. cfNOMe performs a multimodal analysis by complementing methylation with nucleosome occupancy profiles. To account for incomplete reference atlases, CelFiE (Caggiano *et al.* 2021) extended previous approaches by inferring the methylation patterns of unknown cell types from the data through a probabilistic model. MethylCIBERSORT (Filipski *et al.* 2021) is built on support vector regression (SVR) to perform robust deconvolution, discarding the effect of markers with low reconstruction error, whereas ARIC (Zhang *et al.* 2022), also SVR-based, introduces a feature selection step to remove redundant markers, using condition numbers as a measure of collinearity. MethylResolver (Arneson *et al.* 2020), in contrast, alleviates the effect of outliers by using a least trimmed squares approach. CancerLocator (Kang *et al.* 2017) uses a probabilistic model to estimate the tumor burden and identify the correct cancer type, while CancerDetector (Li *et al.* 2018), performs cancer classification at the level of individual sequencing reads. Finally, MeDeCom (Lutsik *et al.* 2017) is a reference-free approach based on regularized matrix factorization. Although each method has its specific merits, it also has its limitations. For instance, neither CancerLocator nor CancerDetector allows full deconvolution of all cfDNA components but solely estimates the cancer proportions, thus limiting the results' interpretability. Moreover, none of the listed methods has been specifically designed to deconvolute multiple cancer tissues. Also, most methods (besides CelFiE) do not consider missing variables due to the incompleteness of the atlas (Abbosh *et al.* 2017, Erger *et al.* 2020) or operate in a reference-free fashion (Lutsik *et al.* 2017). However, cfDNA mixtures are more complex and could carry fragments from tissues not represented in the atlas.

To address these limitations, we developed an alternative reference-based deconvolution method, named MetDecode. We used in-house sequenced samples and complemented them with publicly available tumor samples to build a reference atlas of tissue-specific methylation markers for four different cancer tissues, namely breast, ovarian, cervical, and colorectal cancer, and combined it with white blood cells (WBC)-derived entities. The reference atlas was subsequently exploited by MetDecode to estimate the contribution of each atlas entity in cfDNA samples. In addition, MetDecode can account for missing data by extending the reference atlas with unknown methylation patterns learnt on-the-fly from cfDNA methylation profiles. This method could complement cancer screening programs to direct clinical follow-up to the right cancer type and consequently expedite treatment.

## 2 Materials and methods

### 2.1 Plasma cfDNA and genomic DNA collection and extraction

Peripheral blood was collected in Roche cell-free DNA blood collection tubes^®^ (Roche, Switzerland) or a Streck Cell-Free BCT^®^ (Streck, USA) and extracted as described previously (Lenaerts *et al.* 2019). Archived (Lenaerts *et al.* 2019) and prospectively collected plasma cfDNA samples of healthy individuals were included as control samples (18–90 years old). We included only individuals without cancer or known autoimmune conditions to exclude confounding factors from the analysis, as both pathological conditions can influence the shedding and the composition of cfDNA (Mendioroz *et al.* 2018, Mondelo-Macía *et al.* 2021, Khemka *et al.* 2024). Archived plasma cfDNA was also obtained from patients with a known diagnosis of breast, colorectal or ovarian cancer (mean age: 61.88 years old). Treatment-naïve formalin-fixed paraffin-embedded (FFPE) tumor biopsies were collected. Genomic DNA (gDNA) was extracted from the FFPE tumor biopsies as well as from WBC from healthy subjects or patients with a diagnosis of breast, colorectal, cervical, or ovarian cancer using the QIAamp DNA FFPE Tissue Kit or the DNeasy Blood & Tissue Kits (Qiagen, Hilden, Germany), respectively. The extracted gDNA was fragmented using Covaris M220 before library preparation (Covaris Inc., Woburn, MA, USA). The study was approved by the ethical committee of the University Hospitals Leuven (study protocols S62285, S62795, S63983, S66450, S59207 and S51375).

### 2.2 Complete blood count

Advia 2120 hemacytometer was used to perform the complete blood count (CBC) analysis on whole blood following manufacturer’s instructions.

### 2.3 Whole-genome methylation sequencing and data analysis

cfDNA and gDNA extracted from FFPE tumor biopsies or WBC were subjected to whole-genome DNA methylation sequencing using the NEBNext Enzymatic Methyl-seq kit (New England Biolabs, Ipswich, MA, USA) following manufacturer’s instructions. Enzymatic conversion was preferred over bisulfite conversion for methylation analysis, avoiding fragmentation and loss of DNA in the process (Morrison *et al.* 2021, Vaisvila *et al.* 2021). For gDNA from cervical and ovarian FFPE tumor biopsies that were used to build the reference atlas, bisulfite conversion was performed, to be consistent with the method used for the remaining samples in the atlas. Libraries were prepared with the same kit, thereby replacing the enzymatic conversion reactions with the bisulfite treatment using EZ-96 DNA Methylation-Direct MagPrep (Zymo Research, Irvine, CA, USA). The conversion efficiency was evaluated by spiking unmethylated Lambda DNA in one sample per batch, irrespective of the conversion method used. Libraries were quantified using Qubit dsDNA high-sensitivity assay kit and Qubit 3.0 fluorometer (Thermo Fisher Scientific, Waltham, MA, USA). Libraries were sequenced on NovaSeq 6000 S4 flowcell (Illumina, San Diego, CA, USA) generating PE150bp reads at an average depth of 15X. The data after demultiplexing was quality checked and trimmed using fastp (v0.20) and then aligned to human genome hg38 using bwa-meth (v0.2.2). Deduplication was done using Picard (v2.20.3) and methylation calling via MethylDackel (v0.5.1). The tumor fraction in the cfDNA samples was calculated using ichorCNA (Adalsteinsson *et al.* 2017).

### 2.4 Generation of a DNA methylation marker atlas for multiple blood cell types and tumor tissues

A DNA methylation marker atlas, covering markers for six tumor tissues and seven blood cell types was generated solely using whole-genome bisulfite sequencing (WGBS) data. From public repositories, we downloaded genome-wide CpG site methylation ratios for B cells, CD4+ T cells, CD8+ T cells, natural killer cells, monocytes, neutrophils and erythroblasts [BLUEPRINT (Fernández *et al.* 2016), GSE186458], and for breast invasive carcinoma, colon adenocarcinoma and rectal adenocarcinoma tissues (Supplementary Table S1, Supplementary File S2). WGBS data for high-grade serous ovarian carcinoma, cervical adenocarcinoma and cervical squamocellular carcinoma were generated in-house from FFPE samples. Available samples (*n* = 2–7) per tissue/cell type were merged after removing highly variant CpG sites across replicates, which resulted in a single entity per tissue/cell type (Supplementary File S1). The replicates were aggregated into unique tissue types by summing their CpG counts.

**Figure 1.**
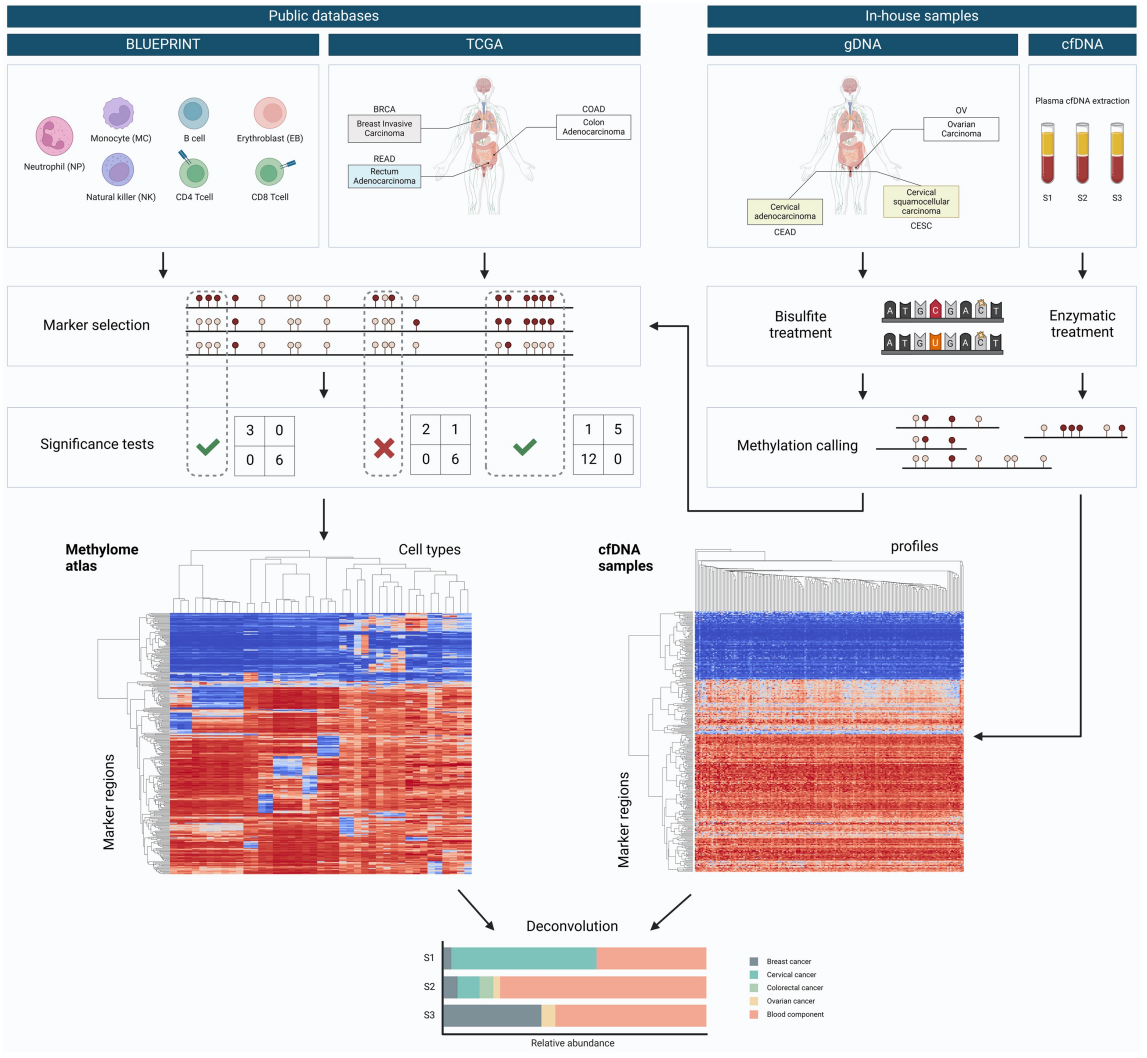
A graphical illustration of MetDecode. Our reference atlas comprises seven blood cell types from BLUEPRINT, three cancer cell types from TCGA, and three in-house cancer types. Unmethylated cytosines from in-house cfDNA and gDNA samples were enzymatically and bisulfite converted, respectively, methylation was called using MethylDackel, and DMRs were determined by extending CpG sites with >30% differential methylation to regions of at least 4 CpGs. As an alternative to the full marker list, a shortlist of statistically significant DMRs was determined using Fisher’s exact test, as well as the list of 23 most significant DMRs per cell type (23×13 = 299 in total). While the full list of markers was used in the main text, results based on the shortlists are presented in Supplementary materials. Finally, an in-house deconvolution algorithm was used to estimate the cell type contributions to cfDNA. The figure was created with BioRender.com.

Differentially methylated sites (DMS), with at least 30% difference between the absolute methylation value in that tissue versus the rest, were extracted using R scripts and extended downstream the CpG site to cover a region with a minimum of 4 CpGs, if the sites were within 500 bp, to define the differentially methylated regions (DMR). The selected DMRs were then filtered based on the minimum region length of 50 bp, 100 bp, and 250 bp. We refer to atlas as “condensed atlas.” We also tested the “full atlas,” where for identified DMRs the cell- or tissue-type specific replicates were used without merging. Next, CpG count matrices were constructed using custom scripts and then used as input for the deconvolution algorithm (Fig. 1). As an alternative to the full marker list, a shortlist of statistically significant DMRs was determined using Fisher’s exact test, as well as the list of 23 most significant DMRs per cell type (23x13 = 299 in total). Contingency tables for Fisher’s exact test were constructed as depicted in Fig. 1, by counting methylated and unmethylated CpGs in the differentially methylated tissue and summing the methylated and unmethylated counts across all remaining cell types (a more formal description is given in Supplementary Materials, Additional File S1). Throughout the manuscript and except for cfDNA simulations, we restricted our analyses to the full atlas and the full marker list, reporting the results of all the possible settings in Supplementary Materials.

### 2.5 Deconvolution algorithm

We created a methylation-based reference atlas, composed of two matrices $D^{\left(\mathrm{atlas}\right)}$ and $M^{\left(\mathrm{atlas}\right)}$, where $D_{jk}^{\left(\mathrm{atlas}\right)}$ is the total CpG count for the atlas entity j and marker region k, and $M_{jk}^{\left(\mathrm{atlas}\right)}$ the corresponding methylated CpG count. Count matrices are constructed as in CelFiE. Because each CpG site can be spanned by multiple reads, it may contribute multiple times to the same count. Therefore, these values must not be confused with read count or coverage. Similarly, we constructed two count matrices $D^{\left(\mathrm{cfdna}\right)}$ and $M^{\left(\mathrm{cfdna}\right)}$, representing the cfDNA mixtures. Our algorithm has been designed to infer a matrix A of cell type contributions, where $A_{ij}$ is the estimated proportion of cell type j to cfDNA profile i. A was found by minimizing a weighted mean absolute error between the methylation ratios of cfDNA samples and the ratios of convoluted atlas entities. Marker region k in sample i was re-weighted by $\sqrt{D_{ik}^{\left(\mathrm{cfdna}\right)}}$ to better reflect the confidence in the estimation of the methylation ratio $R_{ik}^{\left(\mathrm{cfdna}\right)}$. Weights were normalized so they sum up to one. To address unknown cell types potentially missing from the reference atlas, we inferred them from the cfDNA samples and supplemented the atlas with these estimates. Indeed, when the number of cfDNA samples far exceeds the atlas size, the methylation patterns of these unknown contributors can be learned from the data directly. The assumed number of unknown cell types was defined as a hyperparameter (default = 0). We accounted for the unknown contributors in the cfDNA mixture by appending extra rows to the $D^{\left(\mathrm{cfdna}\right)}$ and $M^{\left(\mathrm{cfdna}\right)}$ matrices. Methylation ratio matrices $R^{\left(\mathrm{cfdna}\right)}$ and $R^{\left(\mathrm{atlas}\right)}$ were computed from the corresponding read count matrices.

$R^{\left(\mathrm{cfdna}\right)}$ was next deconvoluted using nonnegative least squares (NNLS) algorithm and reference matrix $R^{\left(\mathrm{atlas}\right)}$, the residuals were used to define the missing contributor and extend $D^{\left(\mathrm{atlas}\right)}$, $R^{\left(\mathrm{atlas}\right)}$, and $M^{\left(\mathrm{atlas}\right)}\;$by one row each. This procedure was repeated h times to obtain the desired number of unknown contributors h.

Matrix A was next inferred from $R^{\left(\mathrm{cfdna}\right)}$, $R^{\left(\mathrm{atlas}\right)}$, $D^{\left(\mathrm{cfdna}\right)}$, and $D^{\left(\mathrm{atlas}\right)}$ using a gradient-based optimization algorithm (Adam), along with G which represents the methylation ratios of the reference atlas. G is allowed to deviate from $R^{\left(\mathrm{atlas}\right)}$ to compensate for the imperfections of the reference atlas and refine it in a data-driven manner. MetDecode essentially solves a matrix factorization problem, where $R^{\left(\mathrm{cfdna}\right)}$ is approximated by the AG product. After inference, the cell types are re-identified by computing pairwise similarities between the rows of matrices G and $R^{\left(\mathrm{atlas}\right)}$.

A more technical description of the algorithm is provided in Supplementary File S1.

### 2.6 Benchmarking with other deconvolution methods

The benchmarking included the nonnegative least squares linear regression (NNLS) algorithm used in MethAtlas (Moss *et al.* 2018), the quadratic programming (QP) approach used in cfNOMe (Erger *et al.* 2020), CelFiE (Caggiano *et al.* 2021), and CancerLocator (Kang *et al.* 2017), supplied with our in-house developed atlas. For CelFiE, default hyper-parameters were used. However, the number of iterations was reduced to 100 for the simulations presented in Supplementary Figs S4–S6 for computational efficiency. For CancerLocator, we set the parameters to thetaStep = 0.01, logLikelihoodRatioCutoff = 0.000001, and methylationRangeCutoff = 0. The reason for setting the methylation range threshold to 0 is that the tool automatically performs feature selection, which we needed to disable to be able to compare the different algorithms on the same marker set. The likelihood ratio cutoff was set low to reach sufficient sensitivity. Since CancerLocator allows for cancer typing but not for full deconvolution, we included it in the comparison for tumor-of-origin identification, excluding it from the benchmark in other settings, such as relative white blood cell contribution estimation.

### 2.7 Evaluation metrics

Pearson Correlation Coefficient and mean squared error (MSE) were used to evaluate the reliability of MetDecode estimations. We evaluated the accuracy of multiclass cancer TOO prediction as $\frac{\#\left(\mathrm{correctly}\;\mathrm{assigned}\;\mathrm{samples}\right)}{\#\left(\mathrm{total}\;\mathrm{samples}\right)}$. *P*-values were considered significant when below 0.001. In addition, we computed Cohen’s kappa coefficients to account for the multi-class nature of TOO prediction.

To compute the limit of detection of an algorithm, we created 4×10×12 *in-silico* mixtures of tumor gDNA and healthy cfDNA (4 samples, 12 different tumor fractions, 10 replicates), and looked for the tumor fraction at which the algorithm either assigns a 0 contribution or starts misassigning cancer types. For each sample, this limit was averaged across the 10 replicates and different cancer types for more accurate estimation.

### 2.8 Availability of data and materials

The data that support the findings of this study is in controlled access data storage in EGA under EGAS00001007493. MetDecode is available on GitHub as a Python package: Https://github.com/JorisVermeeschLab/MetDecode.

## 3 Results

### 3.1 Creation of a reference atlas via tissue-specific epigenetic marker selection

To enable the deconvolution of a methylome into its potential contributors by assigning the relative proportion to a specific tissue type, a methylation reference atlas with 13 entities was created. We included methylome data from seven cells of hematopoietic origin which are the most represented in plasma cfDNA (Moss *et al.* 2018, Fox-Fisher *et al.* 2021, Loyfer *et al.* 2023), as well as methylome data from six different tumors. The tumor tissues included breast cancer, ovarian cancer, colon adenocarcinoma, rectum adenocarcinoma, cervical adenocarcinoma, and cervical squamous cell carcinoma. These cancers were selected to serve as a proof-of-concept. The seven cell types from the hematopoietic lineage included neutrophils, monocytes, erythroblasts, natural killers, B cells, CD4+ T cells, and CD8+ T cells. Tumor methylome data was downloaded from TCGA for five breast invasive carcinomas from different subtypes (1 luminal A, 1 luminal B, 1 basal-like, 2 HER2), two rectum adenocarcinoma and two colon adenocarcinoma (Supplementary Table S1, Supplementary File S2). Since publicly available data was lacking for cervical and ovarian tumors, we generated genome-wide methylome data for three high-grade serous ovarian carcinomas (HGSOC), two cervical adenocarcinomas and three cervical squamocellular carcinoma in-house (Fig. 1).

**Figure 2.**
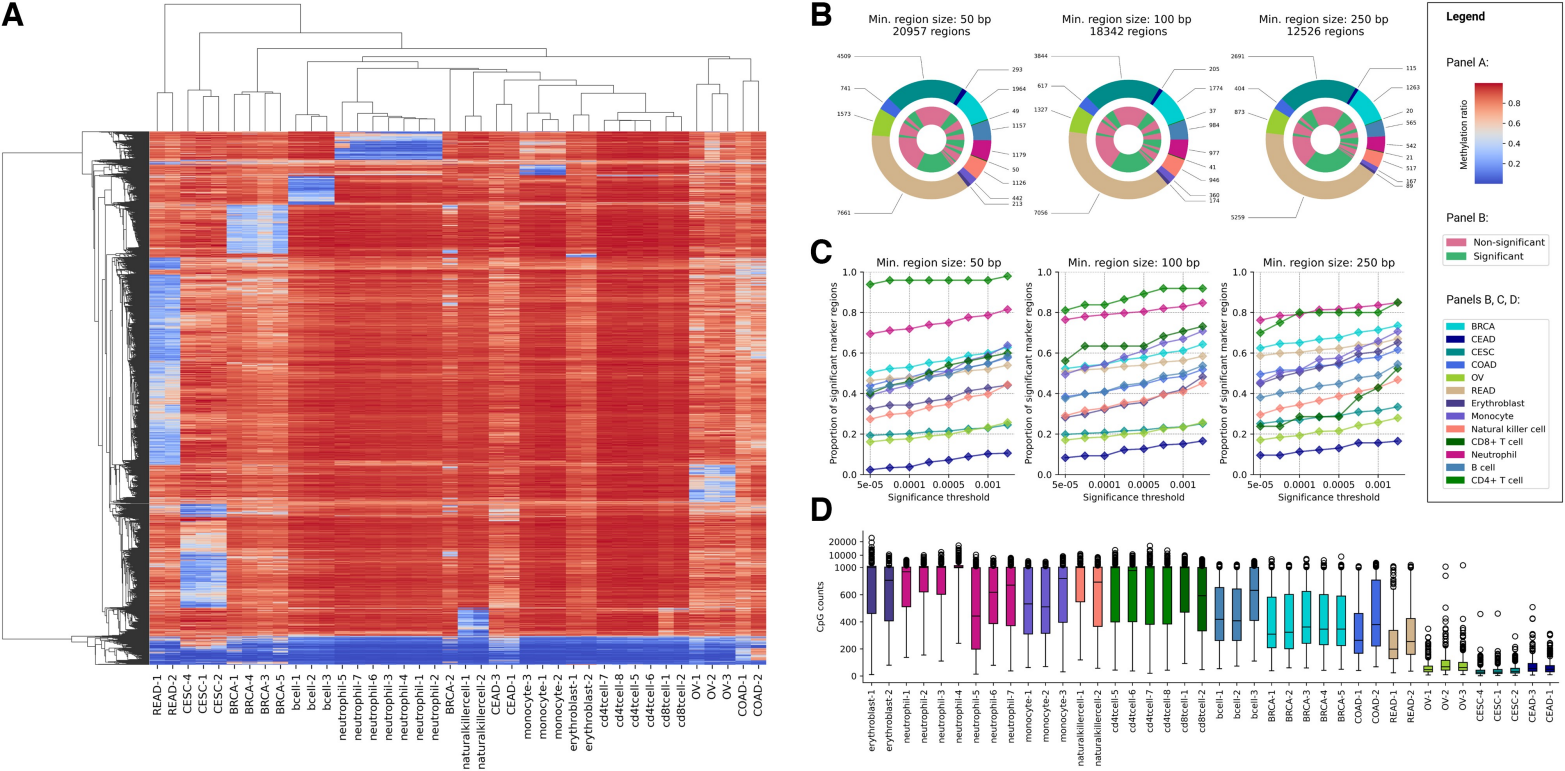
(A) Heatmap displaying the methylation ratios of all the selected marker regions (minimum DMR size of 100 bp) across all atlas entities. The methylation ratio is represented on the colour scale from hypermethylation to hypomethylation. Each sample constituting our full reference atlas is shown on the *X*-axis. (B) Cell-type specific DMR proportions among our marker regions (outer circle) and proportion of significant regions for each cell type (inner circle) across marker sets with different minimum region sizes. We reported the proportion of significant markers for each cell type using a *P*-value cutoff of 0.001. (C) Proportion of significant regions per cell type, as a function of the significance threshold. (D) Distribution of regional CpG counts for each sample in our reference atlas. CpG count was computed as the sum of CpG-level coverages across all CpGs in a marker region. *Y*-axis is linear in the (0, 800) and (800, +∞) ranges. BRCA, breast carcinoma; CEAD, cervical adenocarcinoma; CESC, cervical squamocellular carcinoma, COAD, colorectal adenocarcinoma; OVCA, ovarian carcinoma; READ, rectal adenocarcinoma.

Differentially methylated sites (DMS) were selected by comparing the methylation ratio of each CpG site in one tissue against the other 12 cell/tissue types in the reference (one-versus-all strategy). To ensure that the methylation marker regions were unique, a methylated or unmethylated site was required to have a distinct methylation pattern in one tissue versus the other entities in the reference atlas of minimum 30% (Fig. 2A). Next, CpG sites were extended to regions to ensure sufficient coverage at deconvolution time, enforcing each region to cover a minimum of 4 CpGs and to have a minimum length of 50 bp, 100 bp, or 250 bp. DMS elongation resulted in 20 957, 18 342, and 12 526 marker regions, respectively. The first list had 87.6% and 59.8% of its markers overlapping with the 2 other lists. To account for cell type heterogeneity, variations in methylation margins and variations in coverage, we conducted an exact Fisher test on the CpG counts to assess the significance of the DMRs (Fig. 2B). The choice of significance cutoff (0.00001, 0.00005, 0.0001, 0.0005, 0.001, 0.005, 0.01, or 0.05) did not strongly affect the proportions of significant markers (Fig. 2C). Using a cutoff of 0.001 and Bonferroni correction, we obtained 4572 (50 bp), 4283 (100 bp), and 3372 (250 bp) significant DMRs. The first list had 92.8% and 71.4% of its markers overlapping with the 2 other lists.

A strong imbalance in the number of informative DMRs was observed between reference cell/tissue types. For instance, rectum adenocarcinoma (READ) had the greatest number of significant DMRs, which could be explained by the overall hypomethylation of the READ samples (53.5%). On the other hand, ovarian carcinoma (OV) and cervical squamous cell carcinoma (CESC) had the lowest numbers of significant DMRs, which could be due to the lower coverage of these samples (Fig. 2D). This contrast poses an additional challenge that we tried to tackle while designing our deconvolution algorithm. We tested 3 different deconvolution settings: using (i) all marker regions, (ii) only significant marker regions (0.001 cutoff and Bonferroni correction), and (iii) using the 23 most discriminative marker regions (sorted by *P*-values) for each cell type, resulting in 299 markers (Fig. 2A), where 23 roughly corresponds to the minimal number of DMRs found for a cell type, namely CD4+ T cells (see Fig. 2B).

Apart from our simulations, as well as an exhaustive comparison of all the possible settings on cfDNA deconvolution is reported in Supplementary Fig. S3 (Supplementary File S1) and summarised in Supplementary Table S8 (Supplementary File S2), all the results presented here are based on the full atlas, using all the markers with a minimum size of 100 bp. The *a priori* choice of the full atlas was guided by the aim to account for cancer heterogeneity. We also decided to rely on all marker regions without any filtering, since (i) nonsignificant DMRs can still be more specific to cell types not explicitly listed in the reference atlas, therefore more suitable for the modeling of unknowns, (ii) a comprehensive marker list can help capturing genome-wide methylation changes (e.g. methylation maintenance deficiency in cancer cells), and (iii) the presence of many marker regions can indirectly alleviate the uncertainty caused by the lower coverage of certain atlas entities. The 100 bp cutoff was chosen as a trade-off between 50 and 250 bp.

### 3.2 MetDecode alleviates the effects of noise and unknown contributors

While methylation arrays or targeted sequencing focus on specific loci, whole-genome methylation sequencing produces lower coverage due to reads spanning the entire genome. The coverage reduction per CpG site exacerbates the noise level (Caggiano *et al.* 2021). Not only the genome coverage varies among samples due to sequencing depth differences, but also within the genome itself (e.g. differences in mappability). Accordingly, we devised MetDecode to account for the reliability of methylation ratio estimates in the presence of noise. Since higher coverage of a marker region enables a more accurate estimate of its methylation ratio (assuming the absence of biases), we re-weighted our objective function (see Section 2) to lower the contribution of lower-coverage marker regions in the objective function. The re-weighting does not introduce any bias toward the high-coverage samples (i.e. white blood cells) used to build the atlas, since samples correspond to the rows of the count matrices, while we aim at re-weighting the columns of these matrices instead.

**Figure 3.**
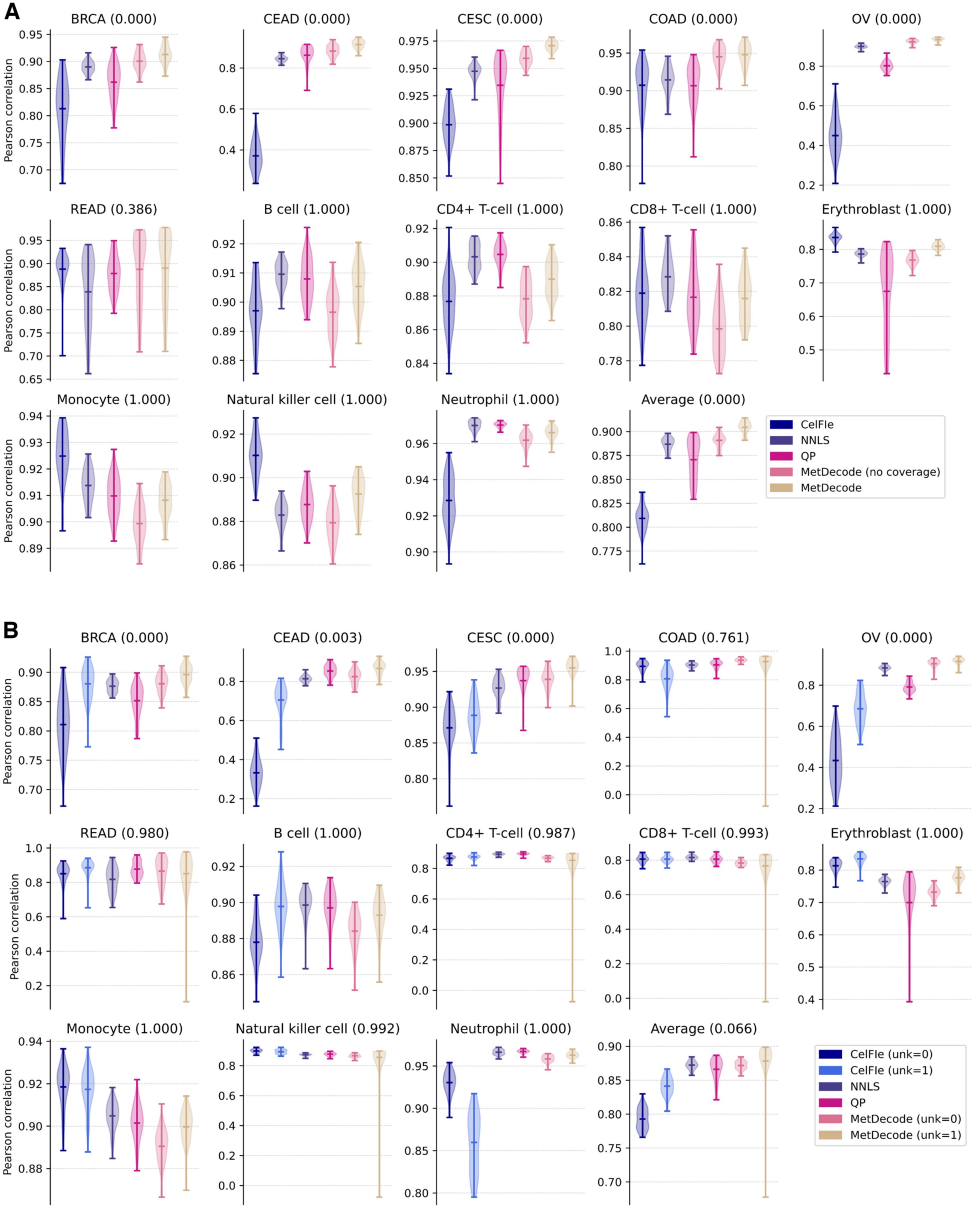
(A) Evaluation of the coverage-based weighting used in MetDecode. We computed Pearson correlation coefficients of different deconvolution algorithms using the shortlist of 23×13 markers with 50 bp minimum length across 50 simulation runs, each containing 5000 simulated cfDNA samples. For each cell type, a one-sided *T*-test has been performed to assess the difference between “MetDecode**”** and the best out of the four other methods: “CelFiE,” “NNLS,” “QP,” and “MetDecode (no coverage).” The corresponding *P*-values have been reported between brackets. (B) Evaluation of the unknown modeling approach used in MetDecode. We computed Pearson correlation coefficients without (unk = 0) and with one unknown (unk = 1) modeled by MetDecode. The *P*-values have been derived from a *T*-test between MetDecode (unk = 1) and the best contender, similarly to the upper panel. BRCA, breast carcinoma; CEAD, cervical adenocarcinoma; CESC, cervical squamocellular carcinoma; COAD, colorectal adenocarcinoma; OVCA, ovarian carcinoma; READ, rectal adenocarcinoma.

To assess the relevance of this new feature for improving deconvolution accuracy, we compared our method with and without the coverage-based weighting approach. For this purpose, we designed simulations based on real data (Supplementary Fig. S1, Supplementary File S1) with random noise injection based on binomial distributions and deconvoluted these random mixtures. In our simulations, cfDNA mixtures were defined as random linear combinations of biased atlas entities, with proportions sampled from a Dirichlet distribution (Supplementary File S1). Due to the number of simulation repeats and for computational speed purposes, simulations were based on the condensed atlas, a minimum marker region size of 50 bp, and limited to the shortlist of 23×13 DMRs. We compared MetDecode against other deconvolution approaches, including MetDecode with disabled weighting strategy. The correlation was significantly higher (*P* < 0.001; *T*-test; one-sided) for all cancer components using the weighting strategy, but not for the blood cell types (Fig. 3A). When averaging the correlation coefficients across all cell types, Pearson correlation for MetDecode was significantly higher than all other approaches (*P* < 0.001; *T*-test; one-sided), therefore highlighting the gain in deconvolution performance obtained when increasing the attention of our deconvolution algorithm on high-coverage regions.

Along with fragments derived from cancerous cells, plasma cfDNA samples also contain contributors from other cell types, mainly from the hematopoietic lineage. A reference atlas of tumors and immune cells will often be incomplete and hence the analysis risks being confounded by unknown contributions. To account for the incompleteness of the atlas, we opted for a data-driven approach that infers the unknown cell types using the cfDNA samples as well as the (incomplete) atlas, based on the residuals obtained after a first round of deconvolution. More specifically, the unknowns are estimated from the difference between the original and reconstructed cfDNA samples, and further refined by gradient descent. To demonstrate the relevance of this novel feature, we performed experiments analogous to those described above to quantify the performance characteristics of MetDecode when modeling one unknown cell type (unk = 1), compared to the situation where the atlas is assumed to be complete (unk = 0). The only difference resides in the addition of an unknown entity with random methylation pattern to simulated cfDNA mixture. While MetDecode (unk = 1) significantly outperformed other methods (including MetDecode unk = 0) on 4 of the 6 cancer components (*P* < 0.001; *T*-test; one-sided, Fig. 3B), but not on the other cell types, the averaged Pearson correlation coefficients were slightly higher for Metdecode (unk = 1), as well as compared to other methods (Fig. 3B). These results highlight the relevance of unknown modeling when unknown cell types in the sample of interest have their methylation patterns loosely correlated with the known atlas entities. The performance gain of CelFiE (unk = 1) over CelFiE (unk = 0) further supports this idea.

We also show the increasing difficulty of deconvolution when the number of unknowns increases and highlight the performance drop of CelFiE when the number of modeled unknowns is larger than needed (Supplementary Figs S4 and S5, Supplementary File S1). In addition, we evaluated the accuracy of MetDecode and CelFiE for the estimation of unknown cell type proportions by individually removing each entity from the atlas (Supplementary Fig. S6, Supplementary File S1). Both algorithms were tasked to model exactly one unknown. We showed the higher Pearson correlation for MetDecode on all cell types, except natural killer cells and neutrophils, therefore demonstrating MetDecode’s higher ability to accurately infer missing components from the cfDNA data directly.

### 3.3 MetDecode allows accurate deconvolution of in-silico mixes

**Figure 4.**
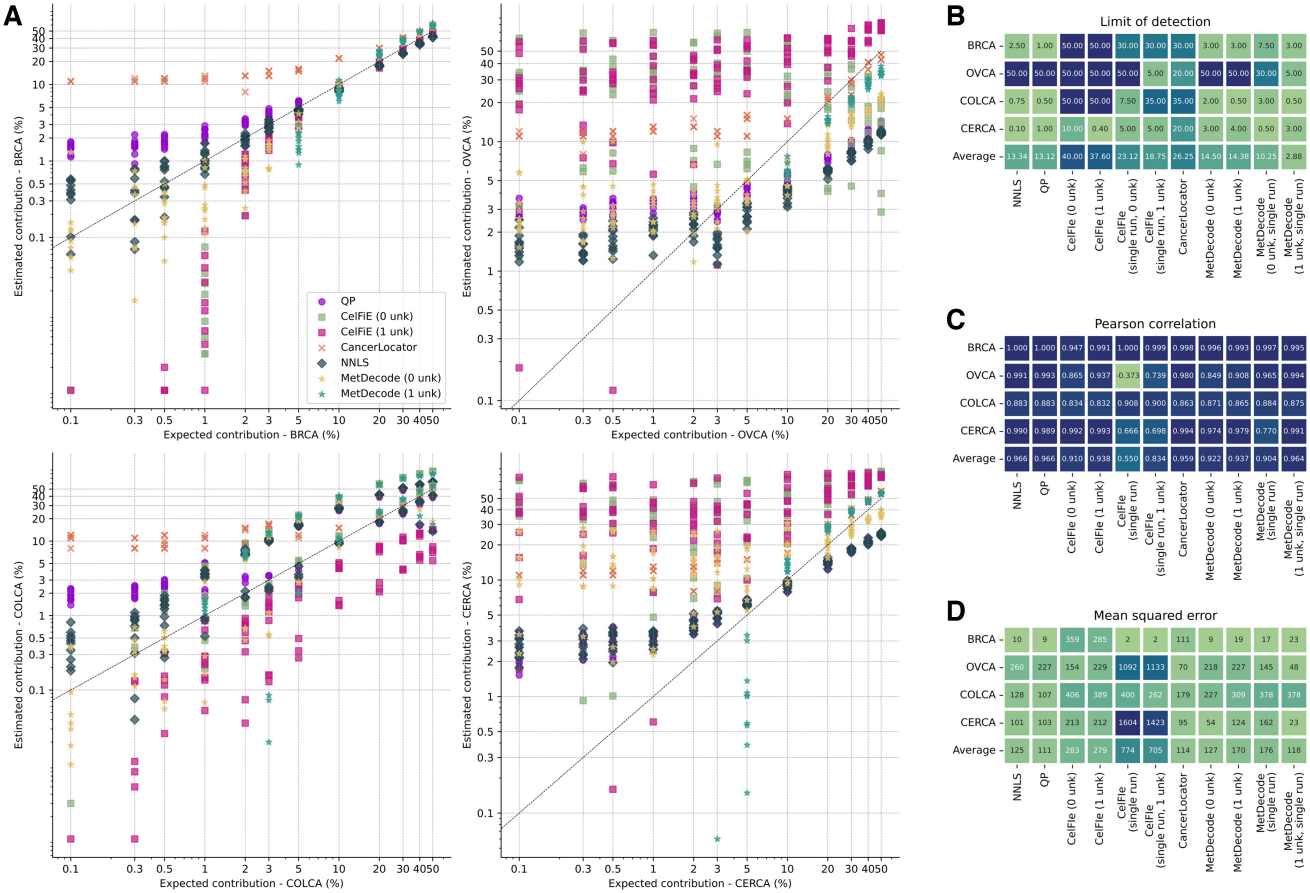
Deconvolution of *in-silico* mixtures using the full atlas and a minimum marker region size of 100 bp. (A) Estimated and ground-truth cancer contributions in percentage of the tumor tissue spiked-in into the *in-silico* mixes. Random reads were combined from a healthy control BAM file and a tumor gDNA BAM file to create an *in-silico* mix and repeated 10 times to obtain replicates. Each dot represents the value for a replicate with a deconvoluted % (*y*-axis) versus expected % (*x*-axis) of tumor tissue DNA. (B) Limit of detections, (C) Pearson correlation coefficients and (D) mean squared errors, averaged across replicates. Because both MetDecode and CelFiE allow modeling of unknowns and partially infer the atlas from the cfDNA data, both methods were run in the same settings: with/without unknown components, and with/without running the tool on all samples at once. BRCA, breast carcinoma; CERCA, cervical carcinoma; COLCA, colorectal carcinoma; OVCA, ovarian carcinoma.

We next evaluated the limit of detection and assessed the concordance in the relative contribution estimation of MetDecode using *in-silico* mixtures of tumor DNA. Breast, colorectal, cervical and ovarian cancer data was combined with cfDNA from healthy donors at ratios varying from 50% to 0.1% to reflect an average depth of 6X (Supplementary File S1). Since CelFiE and MetDecode infer their parameters from the input samples, both algorithms should in principle benefit from larger cohorts. Therefore, we tested both (i) deconvoluting samples of one cancer type separately, and (ii) deconvoluting all samples before reporting the results for each cancer separately. When running on all samples together and using 1 unknown (1 unk, single run), deconvolution of *in-silico* mixes showed that MetDecode detected tumor DNA proportions down to 2.88% on average (Fig. 4A and B), while among other tested methods the best limit of detection (LOD) was 13.12% observed for QP. Running MetDecode in the first setting (one sample type at a time) produced a lower performance in terms of LOD, therefore highlighting the importance of providing the tool with diverse and numerous input samples. The mean correlation between the expected and the estimated percentages was *r* = 0.964 (*P* < 0.001 using a permutation test) for MetDecode (1 unk, single run), which was almost on par with NNLS and QP (Fig. 4B, *P* = 0.966). The highest correlation for MetDecode (1 unk, single run) was obtained for the breast cancer *in-silico* mixes, followed by ovarian, cervical, and colorectal (Fig. 4C). On average, MetDecode produced a MSE of 118, close to QP (111), and CancerLocator (114; Fig. 4D).

While the high LOD in other methods was primarily due to cancer-type misassignments, the LOD of MetDecode (1 unk, single run) was determined solely by its sensitivity. MetDecode mostly assigned the correct cancer types but began to assign zeroes below a certain tumor fraction. In conclusion, MetDecode performed better when applied to all available samples with the unknown modeling enabled.

### 3.4 MetDecode identifies the correct TOO in genomic DNA from leukocytes and tumor tissue

To evaluate the accuracy of MetDecode for deconvoluting and assigning the correct tissue type, we applied the method on 12 WBC-derived gDNA methylomes from healthy controls (mean age: 48.08 years; range: 22–77; M/F: 5/7) and 20 gDNA methylomes from tumor tissue biopsy of breast (*n* = 5), colorectal (*n* = 6), cervical (*n* = 6), and ovarian (*n* = 3) cancer (Supplementary Table S3, Supplementary File S2). These samples were independent from the samples used to create the reference atlas.

**Figure 5.**
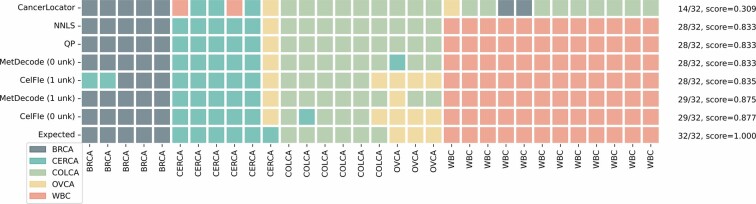
Classification of gDNA samples from WBC and tumor FFPE biopsy based on the cell proportions estimated by different deconvolution algorithms, using the full atlas with all marker regions and minimum region size of 100 bp. The actual tissue type is reported in the last row of the table. Rows have been sorted by increasing Cohen’s kappa coefficient. Accuracy and Cohen’s kappa coefficient have been reported for each row, right to the grid. BRCA, breast carcinoma; CERCA, cervical carcinoma; COLCA, colorectal carcinoma; OVCA, ovarian carcinoma.

When the major contributor amongst all the atlas entities was the expected tissue, the assignment was considered correct. Being the neutrophils the most prominent cells in the blood (Rosales 2018), the deconvolution of genomic DNA extracted from blood was considered as correctly assigned when neutrophils were estimated as the main contributor. In the absence of unknown modeling (0 unk), MetDecode assigned the correct tissue in 28 out of 32 samples, on par with most other methods, except CancerLocator which performed significantly worse (Fig. 5). When adding one unknown component to MetDecode, 29 samples were correctly classified (Overall Accuracy: 90.63%), therefore supporting again the added value of unknown modeling (Supplementary Table S4, Supplementary File S2). Finally, CelFiE (1 unk), QP, and NNLS performed identically (Fig. 5). CelFiE (0 unk) was on par with MetDecode (1 unk).

All WBC-derived samples showed neutrophils as the main contributor, being correctly classified (Fig. 5). All five breast tumor samples and six colorectal samples were assigned to their respective cancer. In addition, 5/6 cervical and 1/3 ovarian tumors were classified correctly. Interestingly, among the correctly classified cervical cancers (5/6), 2/3 CESC and 2/2 CEAD were assigned to the correct subtype category, suggesting the importance of subtype representation in the atlas. One of the six cervical samples was classified as an ovarian tumor consistently across the methods and settings. In addition, two out of three ovarian tumor samples (2 clear cell carcinomas) were misclassified as colorectal cancers (COAD).

**Figure 6.**
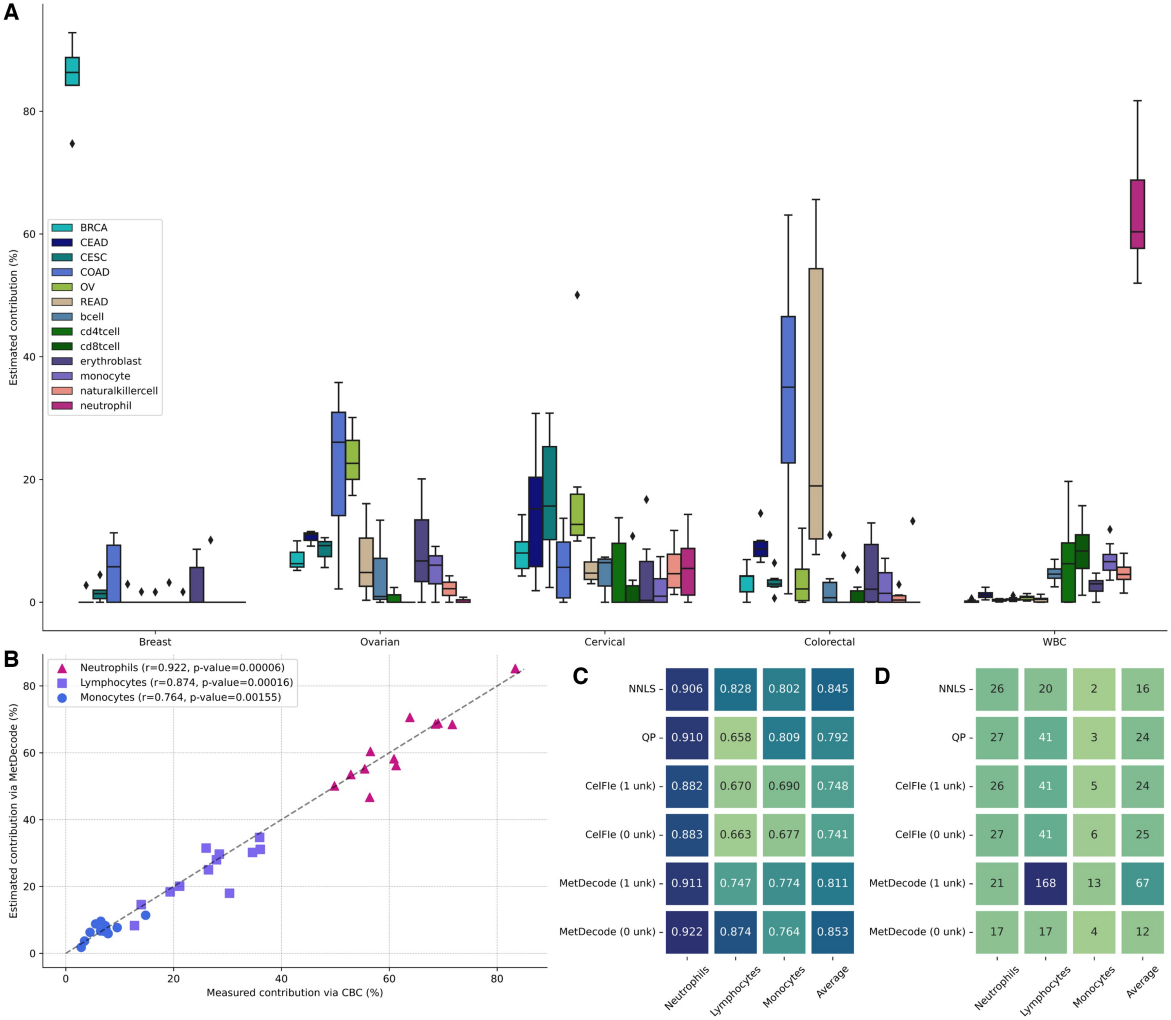
Deconvolution of genomic DNA methylation profiles using full atlas and a minimum marker region size of 100 bp. (A) Deconvolution of gDNA derived from WBC and tumor FFPE biopsies using MetDecode. For each sample group, the estimated contribution from every atlas entity is shown. Each box represents the interquartile range, the extremities represent the minimum and the maximum value. The median is marked with a horizontal bar inside the box. Outliers are depicted by black diamonds. WBC, white blood cells; BRCA, breast carcinoma; CERCA, cervical carcinoma; COLCA, colorectal carcinoma; OVCA, ovarian carcinoma. (B) Relative contribution measured via CBC (*X*-axis) versus the proportion estimated using MetDecode (*Y*-axis) for neutrophils, lymphocytes and monocytes in the gDNA derived from WBC. The diagonal line represents the identity line. *P*-values are based on a permutation test with 100 000 permutations. (C) Pearson correlation coefficients and (D) mean squared errors of different deconvolution algorithms. Ground-truth proportions of neutrophils, monocytes and lymphocytes are based on complete blood count proportions (CBC).

Next, to assess the accuracy of complex mixture deconvolution, we compared the results of the WBC deconvolution to Complete Blood Counting (CBC) of the same blood samples. We observed a high correlation for the neutrophils fraction (*r* = 0.922; *P*-value < 0.001; permutation test, Fig. 6B) when comparing CBC and MetDecode deconvolution estimates. Lower correlation was found for lymphocytes and monocytes (*r* = 0.874; *r* = 0.764, respectively). MetDecode (0 unk) outperformed the best contender (NNLS) in terms of average Pearson correlation (MetDecode: 0.853; NNLS: 0.845, Fig. 6C) and average MSE (MetDecode: 12; NNLS: 16, Fig. 6D). However, the improvement of MetDecode over NNLS was not deemed significant (*P* = 0.0925; one-sided *T*-test on the squared residuals), probably due to the small number of data points (3×12). Overall, these results demonstrate that MetDecode can accurately estimate proportions in samples containing a mixture of cell types.

### 3.5 MetDecode correctly identified the tumor origin in cfDNA from cancer patients

MetDecode was subsequently applied to whole-genome cfDNA methylation sequencing data from healthy controls (93 samples; mean age: 66.8 years; age range: 18–90) and treatment-naive patients with a confirmed cancer diagnosis (93 samples; 41 breast, 13 ovarian, 4 cervical, and 35 colorectal cancers; Supplementary Table S5, Supplementary File S2).

**Figure 7.**
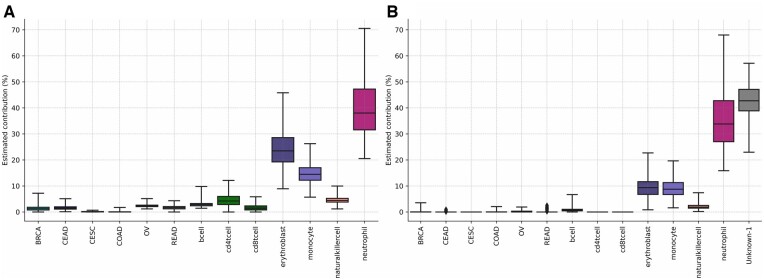
Cell type proportions estimated by MetDecode on 93 healthy controls (A) without modeling of unknowns and (B) with 1 unknown. Each box represents the interquartile range, and the extremities represent the minimum and the maximum value. The median is marked by a horizontal bar inside the box. Black diamonds are used to mark outliers. BRCA, breast carcinoma; CERCA, cervical carcinoma; COLCA, colorectal carcinoma; OVCA, ovarian carcinoma.

cfDNA data from the healthy individuals was used as control to establish the reference range of each atlas entity (Supplementary Table S6, Supplementary File S2). As expected (Moss *et al.* 2018, Fox-Fisher *et al.* 2021), neutrophils were the major contributors to plasma cfDNA (36.7% ± 11.5), followed by erythroblasts (24.0% ± 8.1), and monocytes (14.8% ± 4.8) (Fig. 7A). When adding one unknown to the model, the observed trend remained unchanged, but the CESC and CD4/8+ T cell contributions were entirely transferred to the unknown components (Fig. 7B), and the contributions from other cancer types were strongly attenuated. While the decrease in cancer contributions can be reasonably expected (healthy controls), the drop in CD4/8+ T cell estimates can more likely be attributed to overfitting. The trade-off introduced by unknown modeling between sensitivity and the accuracy of cancer typing was already observed for *in-silico* mixes (Fig. 4A and B).

**Figure 8.**
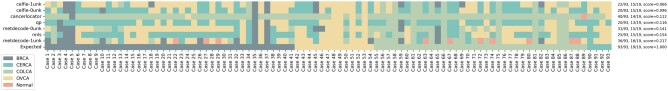
Cancer type prediction by different deconvolution algorithms based on the highest cancer contributors. Ground-truth cancer types have been reported in the last row. Rows have been sorted by increasing Cohen’s kappa coefficient. Right to the grid, each row has been annotated by the accuracy on all cancer cases, the accuracy on the 19 samples with tumor fraction >3% [according to ichorCNA (Adalsteinsson *et al.* 2017)] and Cohen’s kappa coefficient. The cutoff of 3% roughly corresponds to the limit of detection of MetDecode based on *in-silico* mixes (2.88%).

We next assessed the ability of MetDecode and other deconvolution algorithms to assign the correct TOO in these cfDNA samples from cancer patients. We used the controls to normalize the estimated proportions in cancer cases. For each method and each cell type, we divided the estimated proportions by the maximum value across the 93 controls. The highest normalized contributor across the cancer components of the reference atlas was regarded as the putative TOO of the malignancy. When a method estimated each cancer contributor as zero, the predicted class was assumed “normal.” MetDecode (1 unknown) produced the best performance (based on Cohen’s kappa), with a Cohen’s kappa coefficient of 0.217 and 36/93 correctly assigned cancers. We observed the correct assignment of 10/41 breast, 15/35 colorectal, 10/13 ovarian, and 1/4 cervical cancer samples using MetDecode with 1 unknown (Fig. 8). The only cervical sample correctly classified showed a higher contribution of CEAD component, being indeed a case affected by this particular subtype of cervical cancer. Half of the misclassified samples were classified as ovarian cancers (28/57; Supplementary Tables S5 and S7, Supplementary File S2) (Fig. 8).

We also selected the samples with a minimum tumor fraction (TF) of 3% [measurement based on ichorCNA (Adalsteinsson *et al.* 2017)], roughly corresponding to the average LOD of MetDecode based on the experiments conducted on the *in-silico* mixes (2.88%). Considering the samples with a tumor fraction higher than MetDecode’s LOD, MetDecode assigned the correct TOO in 16 out of 19 cancer cases (accuracy 84.2%). One mucinous ovarian carcinoma (case 45, stage IV) was predicted to have breast cancer tissue as a major cancer contributor. One triple-negative breast cancer sample (case 8, stage I) was misclassified as ovarian.

For completeness, we repeated the cfDNA analysis across all versions of our atlas, marker region sizes and filtering methods (Supplementary Fig. S3, Supplementary File S1). When considering all cancer cases, MetDecode (1 unknown, full atlas, 100 bp, all markers) still performed the best, with a Cohen’s kappa coefficient of 0.218 and correctly classifying 38 out of 93 cases.

Overall, MetDecode outperformed the rest of the methods across all versions of the atlas (full/condensed, different marker region sizes, different marker filters), as reported in Supplementary Fig. S3 (Additional File S1) and Supplementary Table S8 (Additional File S2). In particular, MetDecode (1 unk) outperformed CelFiE (*P*** **=** **1.22e−08; one-sided T-test on the Cohen’s kappa coefficients across all settings), QP (*P*** **=** **2.10e−08), NNLS (*P*** **=** **0.0176), and CancerLocator (*P*** **=** **0.0022). The modeling of one unknown improved the performance of both MetDecode (*P*** **=** **0.0011) and Celfie (*P*** **=** **0.0032). The use of the “balanced” marker list slightly degraded performance (*P*** **=** **0.1038), therefore aligning with a previous benchmark study suggesting that deconvolution performance increases with the number of markers (De Ridder *et al.* 2024). Supplementary Figs S7 and S8 (Supplementary File S1) further support the idea that the removal of cell type-specific markers has little consequence on final performance (Supplementary Table S8, Supplementary File S2).

**Figure 9.**
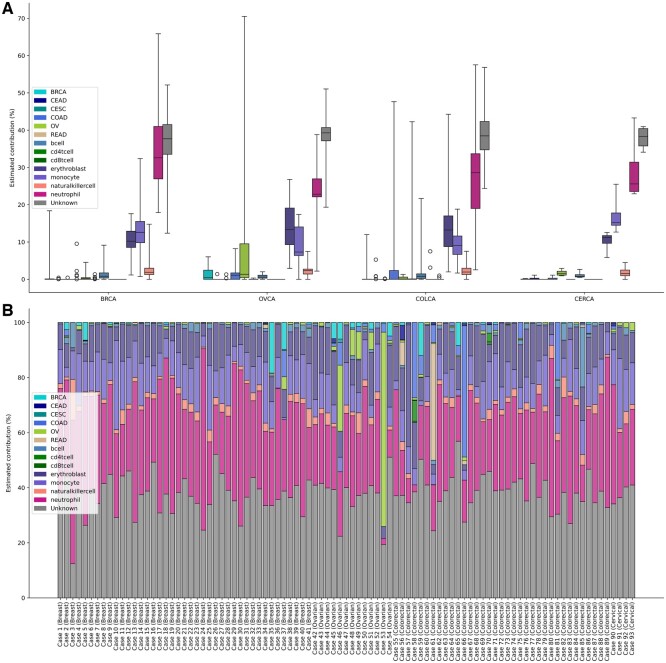
Deconvolution of the cfDNA derived from patients with a confirmed cancer diagnosis using MetDecode. (A) Estimated cell type contributions in each cancer case. For each group of the cohort, namely breast, ovary, and colorectal, the deconvoluted contribution of each atlas entity is represented by a box plot. The box represents the interquartile range, the extremity represents the minimum and the maximum value. The median is marked by a horizontal bar inside the box. Circles are used to represent outliers. (B) Distribution of the deconvoluted percentage in the 93 cfDNA samples from cancer patients. BRCA, breast carcinoma; CERCA, cervical carcinoma; COLCA, colorectal carcinoma; OVCA, ovarian carcinoma.

**Figure 10.**
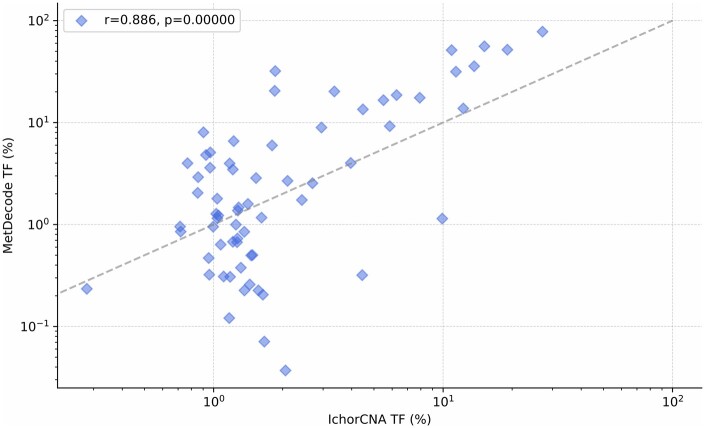
Estimated tumor fraction according to (*X*-axis) ichorCNA and (*Y*-axis) MetDecode using 1 unknown, the full atlas, a minimum marker region size of 100 bp and all marker regions.

Finally, we summed the cancer proportions reported in Fig. 9 across all cancer types and compared them against the tumor fractions estimated by ichorCNA (Adalsteinsson *et al.* 2017). MetDecode attained a Pearson correlation coefficient of 0.886 (*P*** **<** **0.001; permutation test), therefore suggesting that the tool is also relevant for tumor fraction estimation (Fig. 10).

## 4 Discussion

Here, we present a novel method for methylation-based cfDNA deconvolution to aid the identification of cancer types. MetDecode combines a methylome reference with a novel algorithm which can model for unknown contributors absent in the reference and is mindful of the coverage of each marker in the reference. In addition to accurately estimating the tumor proportion in *in-silico* mixtures (*r* = 0.964, *P* < 0.001), the method also assigned the correct TOO in 84.2% (16/19) of the cfDNA samples from cancer patients with TF above MetDecode’s LOD. Overall, MetDecode outperformed other deconvolution methods across all settings in cfDNA samples (CelFiE: *P* = 1.22e−08; QP: *P* = 2.10e−08; NNLS: *P* = 0.0176; CancerLocator: *P* = 0.0022) and benefited from the addition of one unknown entity within its model (*P* = 0.0011).

The performance of CelFiE and CancerLocator remained low across all our experiments, suggesting that these probabilistic models suffered from the lower coverage of some of our atlas entities. Potentially, the underlying statistical assumptions in CelFiE and CancerLocator might be invalidated by the input data. For example, accounting for the coverage of marker regions (e.g. through binomial distributions) is irrelevant when the true underlying methylation ratios are biased (e.g. variable cancer purity in the reference atlas, enzymatic versus bisulfite conversion). Accordingly, our algorithmic design choices for MetDecode to account for both noise and biases in the atlas make MetDecode more robust compared to other deconvolution algorithms.

The deconvolution of *in-silico* mixes and WBC affirmed the accurate performance of MetDecode in complex tissue decomposition. Concerning the deconvolution of tumor tissue gDNA samples, two ovarian carcinomas were misclassified as colorectal cancer and a third one, despite a high contribution of OVCA, exhibited also a significant level of the unknown component. We hypothesize that the OVCA misassignment could result from not including different ovarian carcinoma subtypes in the reference atlas (K**ö**bel *et al.* 2008, Doherty *et al.* 2017). The subtype used to build the reference atlas was HGSOC, while the misassigned gDNA test samples were two clear cell ovarium carcinomas and one mucinous carcinoma. In addition, the in-house ovarian cancer samples used to build the reference atlas suffered from lower coverage compared to the other entities. Therefore, it is possible that some of the marker regions that we selected were spurious and not specific to these tissues. However, we did not observe any clear improvement in deconvolution performance by filtering out nonsignificant DMRs.

Similarly to what was observed in the gDNA, we encountered misclassification of ovarian cancers in cfDNA. However, the sample size and distribution across the different stages/subtypes do not allow any significant and definite explanation to justify the misclassification. Alongside the potential presence of common molecular characteristics across cancers despite a different anatomical location (Heim *et al.* 2014, Terekhanova *et al.* 2023), also the specific ability of a tumor to shed DNA in the circulation are factors that need to be considered when interpreting these results. While for the first problem, a refinement of the reference atlas as mentioned above could help in enhancing performance, the latter requires additional investigations on the cfDNA intrinsic biology.

A way to improve the TOO assignment might reside in using cell-type specific methylation data for the reference atlas creation (Loyfer *et al.* 2023). Cell-type specific methylation would ensure more precise deconvolution and offer clearer insight into the origin of the tumor. However, cell-type-based methylation data is not yet available for the cancer tissues of interest. Similarly, making the atlas entities as much representative as possible of the cancer (sub)category under investigation including samples from different subtypes and stages for the marker selection, could potentially improve the identification of the correct tissue of origin in cfDNA samples (Gerstung *et al.* 2020).

Unique to our approach of selecting methylation markers is that we selected regions with a methylation pattern distinct to one cell or tissue type and, depending on the aims of the end user, it can be applied to different tissues or cell types. Existing marker selection approaches seek to maximize the difference between methylation ratio and the median of the ratio across all tissues. This does not necessarily ensure that only one tissue or cell type is differentially methylated compared to the rest of the entities in the reference atlas (Caggiano *et al.* 2021). The limitation of the one versus all approach is that the number of markers may reduce with an increment in the number of atlas entities. In addition, our methylome atlas was built with a limited number of samples per atlas entity.

To account for imperfections in the reference atlas, MetDecode models the unknown contributor from the input data directly. Because cell types are not perfectly delineated and due to the presence of biological confounders, the role played by unknown modeling is not entirely clear. While MetDecode can compensate for cell types missing in the reference atlas (e.g. liver, megakaryocytes), the ones explicitly present in the reference atlas can also be partially shifted toward the unknown component due to undesired correlations. In Fig. 4, we showed that MetDecode drastically improves the limit of detection using 1 unknown by ceasing from confusing the cancer types, at the cost of shifting part of the cancer signal to the unknown component (hence the presence of zeros in the estimated proportions). Despite these adverse effects, we claim that a large number of available cfDNA samples should help in accurately estimating these unknown components, as supported by our simulations.

## 5 Conclusions

Deconvolution of the cfDNA epigenetic signatures is an elegant approach to deduce the TOO or cancer type. To estimate the contributions and the type of cancer and other cell contributors in a cfDNA sample, we developed MetDecode, a methylome reference-based deconvolution algorithm. MetDecode can model the unknown contributors unavailable in the reference and account for the coverage of each marker region to alleviate the potential sources of noise.

Despite the limited sample size, the results presented here are encouraging and important for the future of liquid biopsy clinical application. In fact, a tool able to pinpoint the TOO of a malignancy can be used to streamline the diagnostic process in cancer patients. Emblematic cases in which the TOO detection via cfDNA can be of clinical utility are in the detection of cancer-like signals in maternal blood during routine noninvasive prenatal screening and in case of metastatic tumors of unknown primary. Deconvoluting and defining the TOO will aid the oncologists in identifying the tumor and direct treatment, streamlining the diagnostic process, especially in cases in which invasive examinations and radiological investigation are not ideal. Furthermore, if specific immune characteristics of the malignancy could be detected thanks to the blood-derived entities residing in the atlas, an important dowel for the treatment decision of the patient can be added simultaneously to the TOO identification. To conclude, we developed a method for deconvoluting the components of cfDNA enabling detection of cancer origin using tissue-specific methylome information.

## Supplementary Material

btae522_Supplementary_Data
