# Supplementary material for: MetDecode: methylation-based deconvolution of cell-free DNA for noninvasive multi-cancer typing
Source: Bioinformatics. 2024 Aug 23;40(9):btae522. doi: 10.1093/bioinformatics/btae522 (PMC11379469; doi:10.1093/bioinformatics/btae522)
Supplement: btae522_Supplementary_Data [file btae522_supplementary_data.zip › Supplementary file 1.docx]

**Additional material**

*Notation*

A reference methylation atlas is provided in the form of two matrices $M^{\left( atlas \right)} \in\mathbb{N}^{m\times p}$and $D^{\left( atlas \right)} \in\mathbb{N}^{m\times p}$, in which $D_{jk}^{\left( atlas \right)}$ is the number of CpG sites spanned by reads in the marker region $r$ for reference cell type $j$ and $M_{jk}^{\left( atlas \right)}$ is the number of methylated ones. $m$ is the number of atlas entities and $p$ the number of marker regions. Similarly, cfDNA data is supplied as two matrices $M^{\left( cfdna \right)} \in\mathbb{N}^{n\times p}$ and $D^{\left( cfdna \right)} \in\left[ 0, 1 \right]^{n\times p}$, where $n$ is the number of patients. Because each CpG site can be spanned by more than one sequencing read, in practice each site can contribute to the counts more than once.

*Differential methylation analysis*

When the difference in methylation ratios in one cell type was larger than 30% compared to all the other atlas entities, we called a CpG site as differentially methylated. However, after merging downstream CpG sites into marker regions, this 30% cutoff was no longer guaranteed to be satisfied. Moreover, the low coverage of the differentially methylated cell type can be responsible for this observed difference. Therefore, we performed Fisher’s exact test on the candidate marker region to assess its significance using fisher_exact function from the Python package SciPy. For each candidate marker region, we constructed a contingency matrix in the form of[[a,b;c,d]], which we used directly as input to the function. For each candidate marker region $k$, we first identified the differentially methylated cell type, which we denote as $j$. The first row of the contingency table corresponds to the methylated (a) and unmethylated (b) counts of the differentially methylated cell type $j$, thus $a = M_{jk}^{\left( atlas \right)}$and $b = D_{jk}^{\left( atlas \right)} - M_{jk}^{\left( atlas \right)}$. The second row of the contingency table corresponds to all the remaining cell types, which we model as a single pseudo-bulked sample. Therefore, $c = \sum_{l=1, l \neq j}^{m} M_{lk}^{\left( atlas \right)}$ and $d = \sum_{l=1, l \neq j}^{m} D_{lk}^{\left( atlas \right)} - M_{lk}^{\left( atlas \right)}$.

While using the most similar cell type to $j$ to build the second row is a valid option, this would comprise the significance of some of the results due to the presence of some lower-coverage samples in the reference atlas.

***Deconvolution algorithm***

*Overview and modelling*

The methylation ratios of the atlas and the cfDNA samples are computed as $R_{jk}^{\left( atlas \right)} = \frac{M_{jk}^{\left( atlas \right)}}{D_{jk}^{\left( atlas \right)}}$ and $R_{ik}^{\left( cfdna \right)} = \frac{M_{ik}^{\left( cfdna \right)}}{D_{ik}^{\left( cfdna \right)}}$, respectively. The goal of our algorithm is to estimate a matrix $A \in\left[ 0, 1 \right]^{n\times m}$, where $A_{ij}$ is the estimated proportion of cell type $j$in cfDNA profile $i$. Before inference, we add $h$ extra rows to matrix $R^{\left( atlas \right)}$ to model the presence of unknown cell types potentially present in the cfDNA mixtures. $h$ is a hyper-parameter that can be tuned by the end-user.

*Imputing missing values*

To prevent numerical issues related to some of the $D_{jk}^{\left( atlas \right)}$ and $D_{jk}^{\left( cfdna \right)}$ values being equal to zero, we imputed the latter with pseudo-counts. This preprocessing step has been applied prior to using any of the following algorithms: MetDecode, CelFiE, NNLS, QP. Indeed, CancerLocator was excluded due to its native ability to account for missing values. When the total count was zero, the methylation ratio was estimated as the product of the weighted row and column averages, using the counts as weights. Imputed methylation ratios were clipped between 0.01 and 0.99 to avoid skewing the data with extreme values.

*Modelling of unknown contributors*

Let’s denote the *k*-th column of $R^{\left( atlas \right)}$ by $R_{.k}^{\left( atlas \right)}$. We first compute the lower bounds $R_{k}^{\left( lb \right)} = Q\left( 0.4; R_{.k}^{\left( atlas \right)} \right)$ and upper bounds $R_{k}^{\left( ub \right)} = Q\left( 0.6; R_{.k}^{\left( atlas \right)} \right)$ on the atlas values, where $Q$is the quantile function. The initial cell type proportion estimates $\alpha$are obtained by the non-negative least squares (NNLS) algorithm. We quantify the excess of methylation of the reconstructed samples in marker region $k$ as the median residual $e_{k} = median_{i} \left( -R_{ik}^{\left( cfdna \right)} + \sum_{j} \alpha_{ij}R_{jk}^{\left( atlas \right)} \right)$. Intuitively, $e_{k}$ is strictly greater (smaller) than 0 when most of the reconstructed cfDNA samples produced methylation ratios higher (lower) than what can be observed in $R^{\left( cfdna \right)}$. Therefore, $e_{k}$ provides a hint on the information currently lacking from the atlas. The new row of $R^{\left( atlas \right)}$ is defined as follows: its $k$th element will be set to $R_{k}^{\left( lb \right)}$ when $e_{k}$ is positive, and vice versa. Corresponding row in $D^{\left( atlas \right)}$ is simply computed as the median read counts across the samples. Corresponding row in $M^{\left( atlas \right)}$ is determined by the element-wise product of methylation ratios and read counts. Once an extra row has been added to $D^{\left( atlas \right)}$, $M^{\left( atlas \right)}$ and $R^{\left( atlas \right)}$, the whole procedure is repeated until the desired number of unknown contributors is reached.

*Alleviating atlas biases by learning from cfDNA data*

In practice, the reference atlas $R^{\left( atlas \right)}$ may not be suitable for accurate deconvolution of cfDNA samples due to various biases and confounding effects. Typical technical confounders can be caused by changes in library preparation protocols and sequencing pipelines. The difference between bisulfite and enzymatic conversion can also affect the overall methylation of the sample. Biological confounders include, among others, age, sex and whether the donor is smoking or not. For these reasons, we did not factorize $R^{\left( cfdna \right)}$ as a $AR^{\left( atlas \right)}$ product, but rather a $AB$product where $B$ is initialized to $R^{\left( atlas \right)}$ but allowed to deviate from it during inference.

*Objective function*

We define the reconstruction error as the weighted average absolute error between the original matrix of methylation ratios $R^{\left( cfdna \right)}$ and the reconstructed matrix, where the weights are given by $W_{ik} \propto\sqrt{D_{ik}^{\left( cfdna \right)}}$. Weights are normalised to sum up to one. The reconstruction error is formulated as $f\left( A \right) = \sum_{i=1}^{n} \sum_{k=1}^{p} W_{ik}\left| R_{ik}^{\left( cfdna \right)} - \sum_{j=1}^{m} A_{ij} B_{jk} \right|$ and our deconvolution algorithm aims at minimising this objective function by gradient-based optimisation.

*Inference*

Parameters $A$ and $B$ are found by gradient-based optimisation, using the pytorch Python package (which ensures full and automated differentiation) and the Adam optimizer. $A$ and $B$ are optimised separately in a block coordinate descent fashion, each with their own dedicated optimised. Each learning rate is decreased by a 0.9 factor when the update of the corresponding parameter resulted in an increase of the objective function at previous iteration and increased by a 1.015 factor otherwise.

Since the elements of $A$ are positive and its rows sum up to one, $A$ is lying on a multinomial manifold. To ensure that $A$ remains on the manifold while optimising, we instead optimise a matrix $A^{'}$ and apply a SoftMax function on the rows of $A^{'}$ to obtain a matrix $A$ the desired properties. Similarly, the elements of B should stay in the [0, 1] range, which we ensure by freely optimising a matrix $B^{'}$ of same dimensions and applying a Sigmoid function to its elements in a differentiable manner.

*Re-identifying cell types*

Because $B$ is not guaranteed to be in the vicinity of $R^{\left( atlas \right)}$ at the end of the inference process, its rows may no longer correspond to the same cell types. Therefore, estimated proportions $A$ may loosely correspond to the reference atlas. Our proposed solution is to permute the columns of $A$ based on correlations between reference and inferred atlas entities. Proposed algorithm comprises the following steps:

- Compute the matrix of pairwise correlations $C$, where $C_{ij}$ is the Spearman correlation between inferred cell type *i* and reference cell type *j*.
- Initialize a matrix $P = 0$ of same dimensions as $C$.
- Iterate over each row *i* by increasing order of average methylation. For each *i*, repeat the following steps:
  - Identify the column *j* with the highest value $C_{ij}$.
  - Set $P_{ij}$ to 1.
  - Set row $C_{i.}$ to $-\infty$.
  - Set column $C_{.j}$ to $-\infty$.
- Replace $A$ by the $AP$ product.

***Simulation procedure***

In Suppl. Fig. 1, we illustrated the data generation process used in our simulations. To simulate the fact that our atlas is not complete, we generated random methylation patterns by randomly sampling from the atlas: for each marker $k$, the ratio was randomly chosen among the available atlas entities, by randomly choosing one value from column $k$ of $R^{\left( atlas \right)}$. This produces unique methylation patterns while ensuring that the marginal distribution matches the rest of the atlas. The newly obtained row is then added to $R^{\left( atlas \right)}$. To simulate the presence of biological and technical confounders, we added both independent bias terms $U_{jk}$ and sample-specific bias terms $V_{j}$. The biased atlas $R^{\left( biased \right)}$ was then computed as $R_{jk}^{\left( biased \right)} = 0.8 R_{jk}^{\left( atlas \right)} + 0.05 U_{jk} + 0.15 V_{j}$. The methylation ratios of cfDNA samples $R^{\left( cfdna \right)}$ have been generated as random linear combinations $\alpha$ of rows from $R^{\left( biased \right)}$. Each row of $\alpha$ has been randomly sampled from a Dirichlet distribution with the following parameters for each cell type: Cancer=5.0, B cell=3.0938, CD4=8.2576, CD8=3.8222, Erythroblast=1.7946, Monocyte=4.6937, Natural killer cell=2.6914, Neutrophil=29.6514, Unknown=3.0. The cancer proportion of each sample was randomly assigned to one cancer tissue with equal probabilities. To simulate the presence of noise, methylated counts have been randomly sampled from binomial distributions using the coverages from our original data. More specifically, the atlas values were defined by $M_{jk}^{\left( atlas \right)} \sim B\left( D_{jk}^{\left( atlas \right)}, R_{jk}^{\left( atlas \right)} \right)$ and cfDNA values by $M_{ik}^{\left( cfdna \right)} \sim B\left( D_{ik}^{\left( cfdna \right)}, R_{ik}^{\left( cfdna \right)} \right)$.

We used the condensed version of the atlas (13 entities) for our simulations, using a minimum marker region size of 50 bp, and a balanced representation of cell types (13 x 23 = 299 markers).


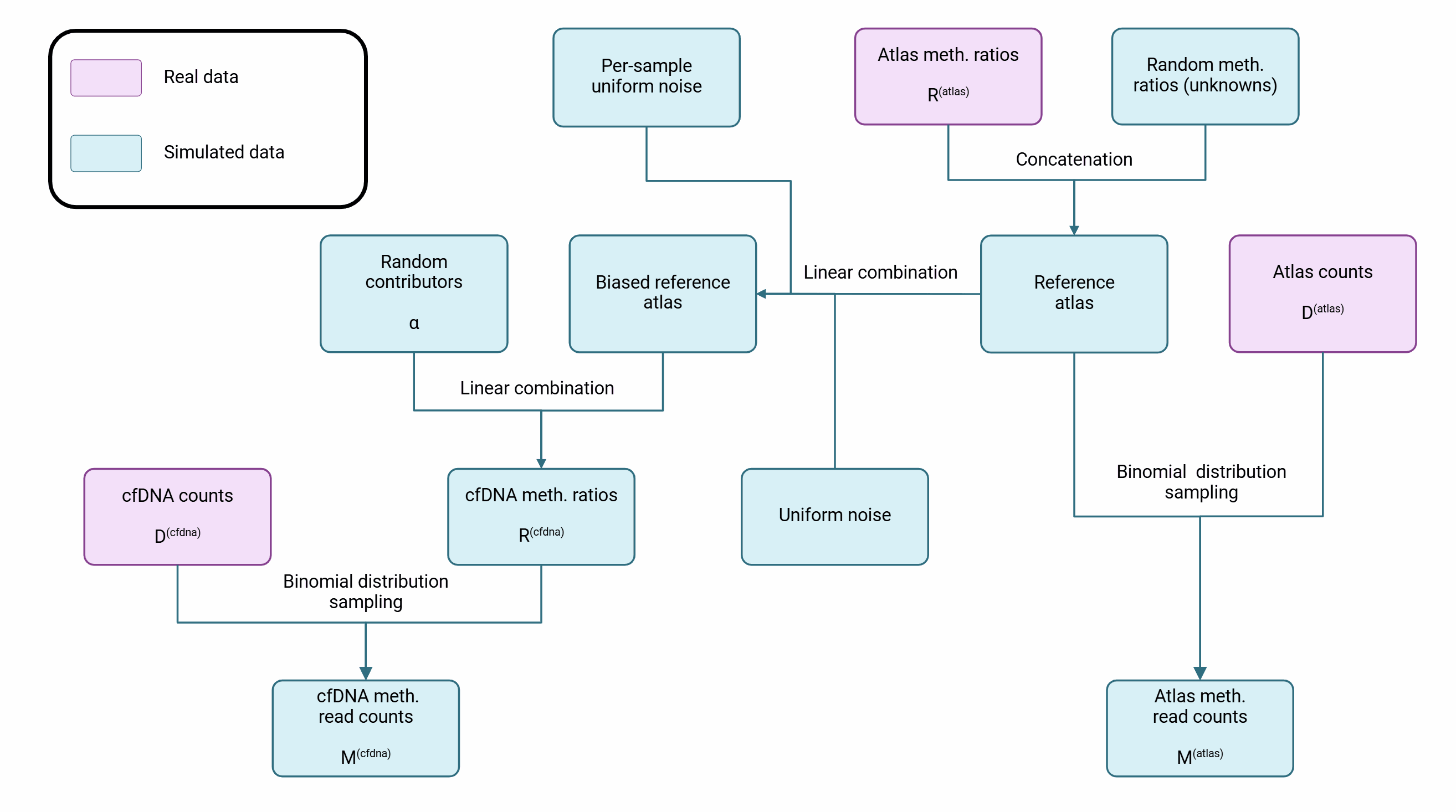
**Supplementary figure 1:** Flowchart of the simulation procedure. We used the counts and methylation ratios from our real atlas to generate the methylation ratios of random cfDNA mixtures, and used the cfDNA counts to determine the coverage of each marker region in these mixtures.

***Sample pre-processing for selecting methylation markers***

In-house methylation data and downloaded data from all the public databases were converted to a uniform 2bp format so that every CpG site is represented in one line. Lift over to hg38 was done where required using in-house scripts. We observed high variance in the methylation ratio of few sites in samples of the same tissue/cell type (Supplementary figure 2). To avoid sample-specific bias, sites with a high variance (var>0.125) were removed. Multiple samples of the same tissue/cell type were then combined by summing up the total reads and methylated reads at each CpG site to improve the coverage. This resulted in a “Combined” file with a methylation ratio of CpG sites per reference atlas entity which was then used for selecting methylation markers.


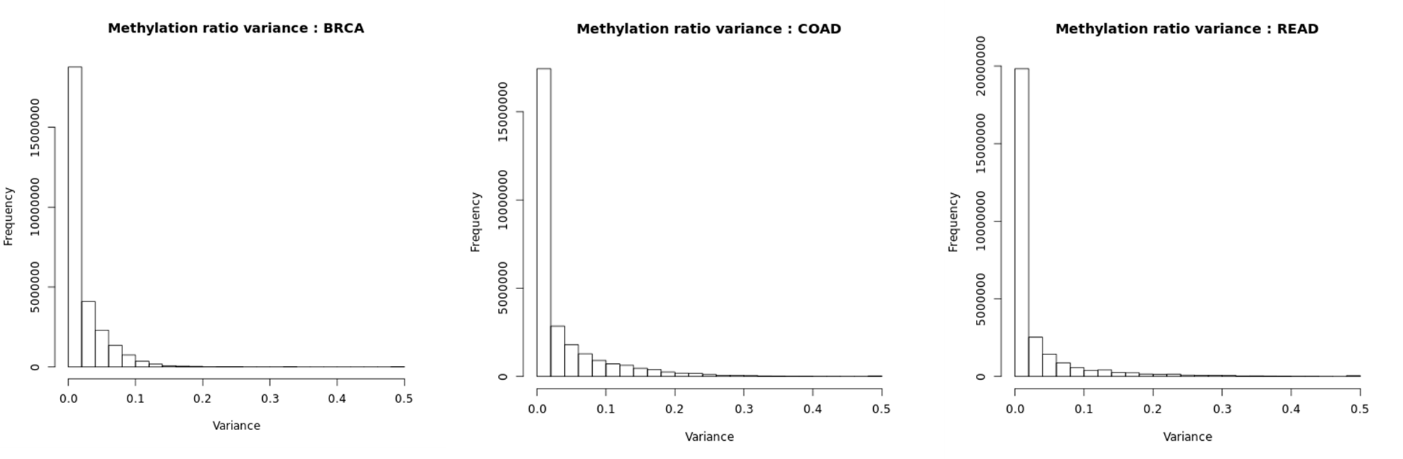
**Supplementary figure 2**: The distribution of variance of methylation ratio at every CpG site across samples from the same tissue type. Tumour samples downloaded from TCGA are shown here as an example. BRCA-Breast invasive carcinoma; COAD-Colon adenocarcinoma; READ-Rectum adenocarcinoma.

***Creation of in-silico mixtures***

*In-silico* mixtures were created using the aligned BAM files from a healthy control cfDNA and a tumour gDNA from a cancer type. The depth of an *in-silico* mix was set to 6X and corresponding read counts from a healthy control cfDNA and tumour gDNA BAM files were calculated based on the 12 tumour fractions (50, 40, 30, 20, 10, 5, 3, 2, 1, 0.5, 0.3 and 0.1). 10 replicates per tumour fraction (120 files in total) were generated by random selection of reads from the BAM file. The pairs of BAM files were then merged using samtools (v1.9) and methylation calling was performed using MethylDackel (v0.5.1). 2 samples from each cancer type namely breast, cervical, colorectal and ovarian were processed in the same manner resulting in 960 BAM files.

***Deconvolution of cfDNA samples from cancer cases***

We ran each deconvolution algorithm (NNLS, QP, CancerLocator, CelFiE and MetDecode) using the full and condensed versions of the atlas. In the condensed version, cell/tissue replicates have been piled up into a single atlas entity, resulting in 13 combined entities in total. Furthermore, each deconvolution algorithm was run with 3 different versions of the base atlas, using minimum marker region sizes of 50 bp, 100 bp and 250 bp, respectively. Finally, 3 different marker region filters have been applied to the atlas (all markers, only the significantly differentiated regions, and the shortlist of 13x23 markers with equal representation of the 13 entities).


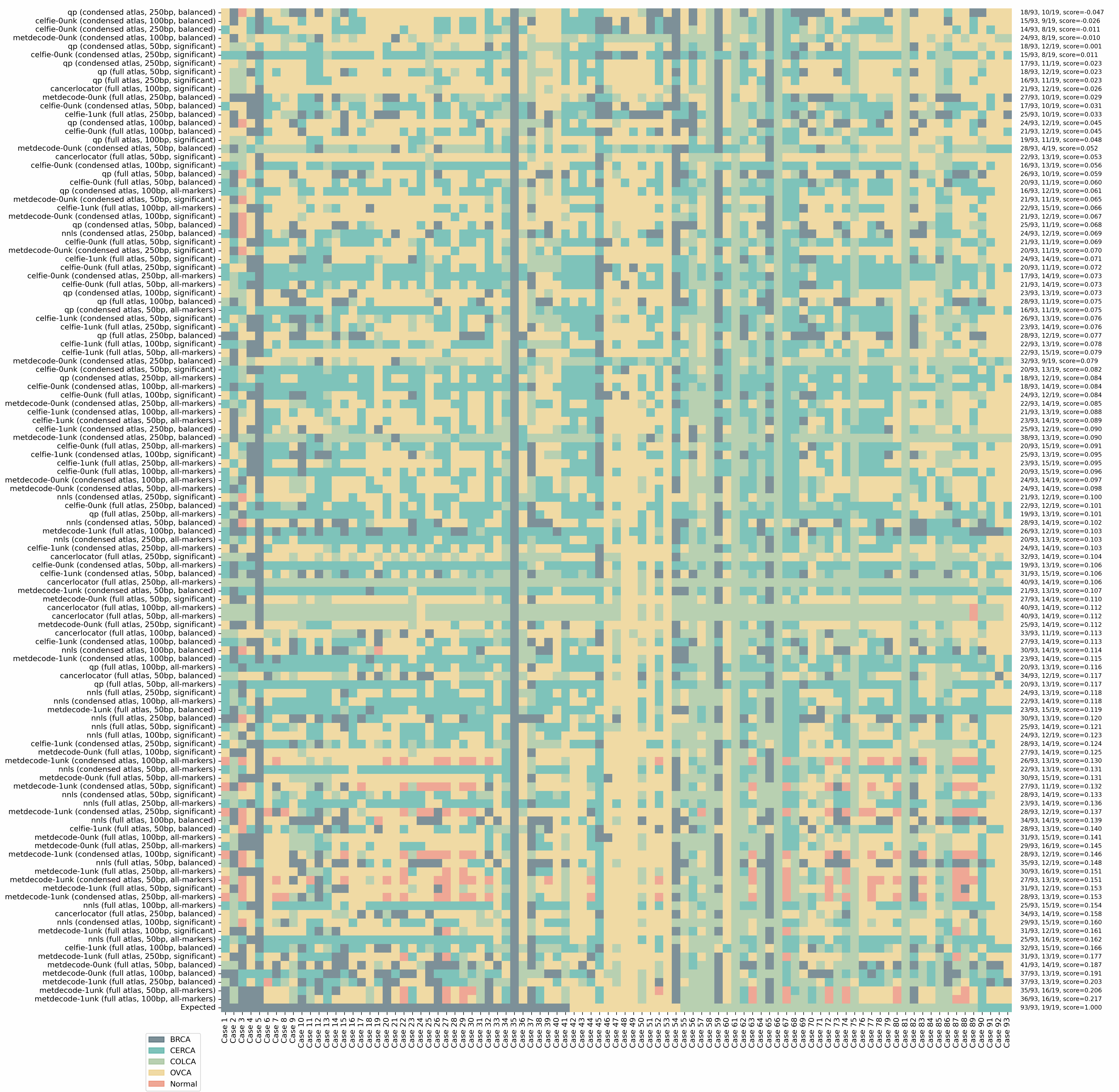
**Supplementary figure 3**: Cancer type assigned by each deconvolution algorithm based on the highest cancer contributors across different experimental settings on all cancer cfDNA cases. ”Full atlas” refers to the full atlas without piling up the replicates/subtypes. ”Condensed atlas” refers to the piled-up atlas with 13 entities. ”50bp“, ”100bp” and ”250” refer to the minimum marker region size used in the atlas. "All-markers", ”significant” and ”balanced” identify the type of filtering applied on the marker list (no filtering, only significantly differentiated regions and shortlist of 299 markers with equal representation for each cell type, respectively). Rows have been sorted by increasing Cohen’s kappa coefficient. Right to the grid, each row has been annotated by the accuracy on all cancer cases, the accuracy on the 19 samples with tumour fraction higher than 3%, and Cohen‘s kappa coefficient. Ground-truth cancer types have been reported in the last row.

Additionally, we conditioned on specific settings and compared the performance between settings (e.g., 50 bp versus 100 bp) using T tests, and reported the results in Supplementary table 8 (Additional file 2).

***Simulations with increasing numbers of unknowns***


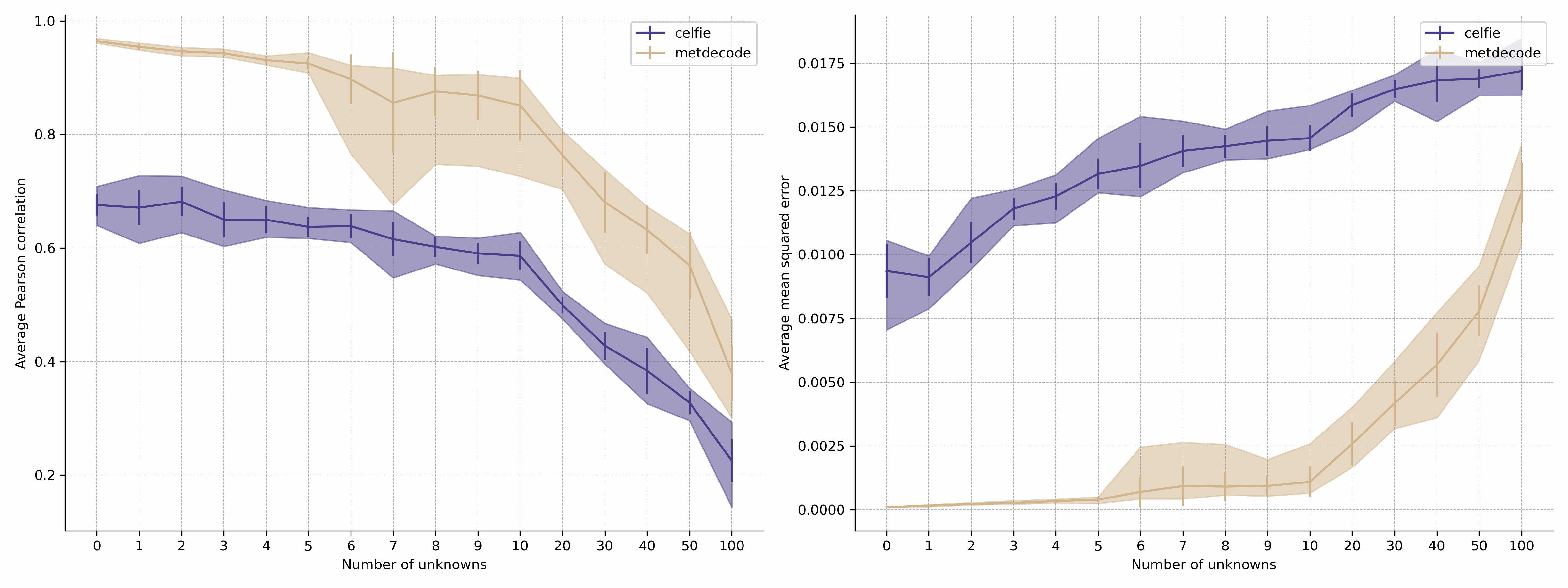
**Supplementary figure 4**: (Left) Pearson correlation and (Right) mean squared error (MSE) of CelFiE and MetDecode when the simulated cfDNA mixtures contain increasing numbers of unknowns, and the number of unknowns within each model is equal to the actual number of unknowns. Shaded area delineates the minimum and maximum values across 10 repeats, and vertical bars represent the standard deviation. Each simulation run produced 100 cfDNA samples. Only the 13 known atlas entities have been used to evaluate the performance metrics. The number of iterations of CelFiE was reduced to 100 iterations for computational efficiency. Simulated methylation ratios were unbiased.


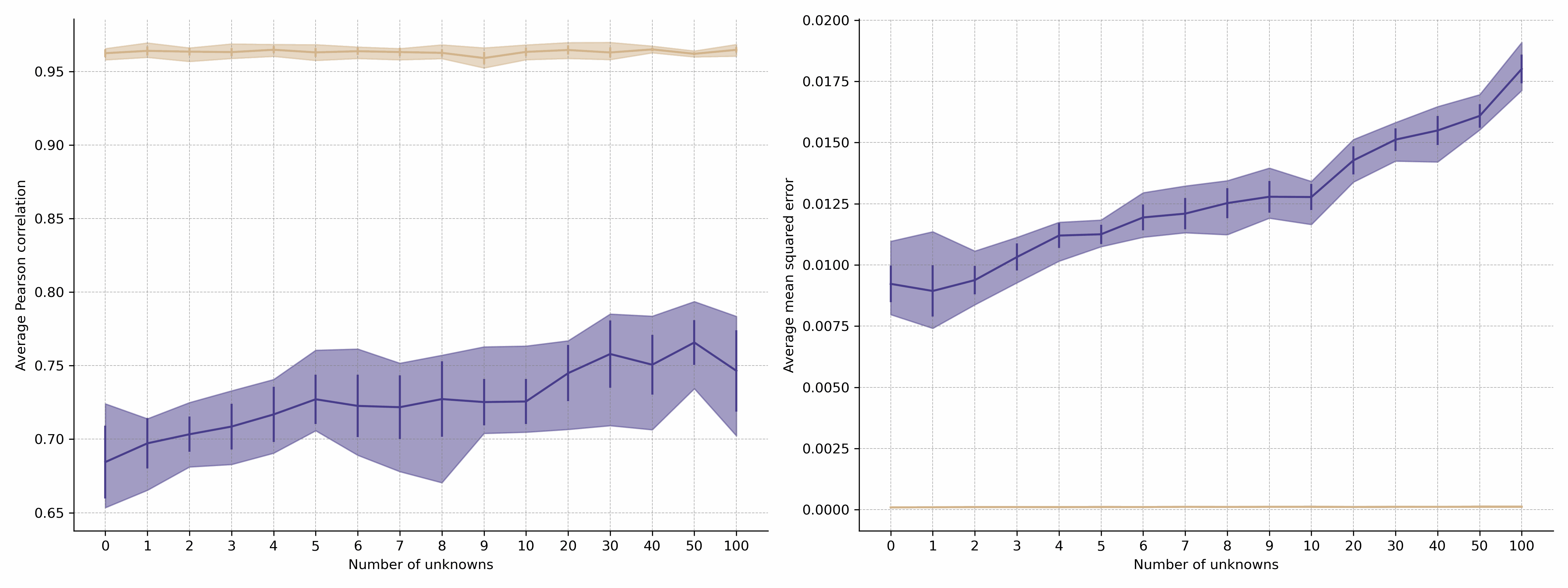
Supplementary figure 5: (Left) Pearson correlation and (Right) mean squared error (MSE) of CelFiE and MetDecode when the simulated cfDNA mixtures contain no unknown (the input atlas is comprehensive), but the number of unknowns within each model is increasing. Shaded area delineates the minimum and maximum values across 10 repeats, and vertical bars represent the standard deviation. Each simulation run produced 100 cfDNA samples. Only the 13 known atlas entities have been used to evaluate the performance metrics. The number of iterations of CelFiE was reduced to 100 iterations for computational efficiency. Simulated methylation ratios were unbiased.

***Simulating MetDecode’s ability to approximate missing cell types***


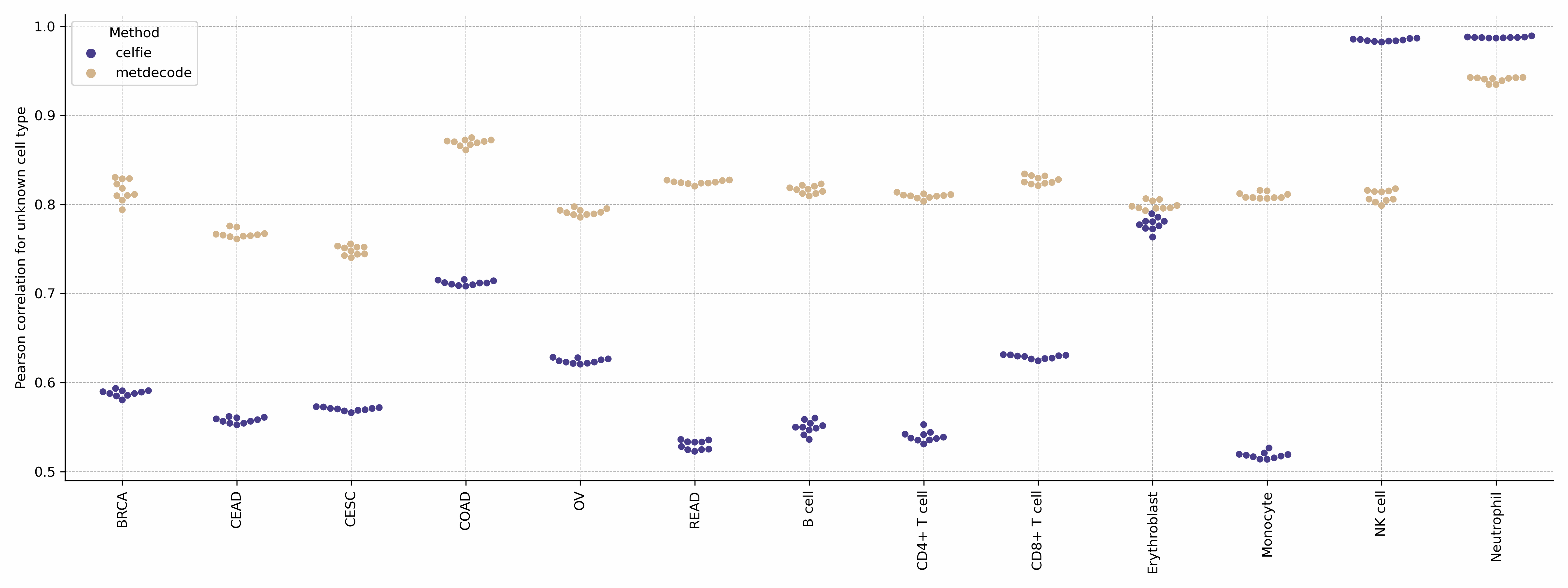
**Supplementary figure 6:** Leave-one-out simulation, where all cell types contribute to the simulated cfDNA samples, but each cell type has been individually removed from the input atlas. Both algorithms were tasked to model exactly one unknown. Mean squared error (MSE) has been computed between the known/ground-truth methylation ratios of the removed atlas entity and the ratios estimated by CelFiE and MetDecode. This simulation has been repeated 10 times. Each simulation run is depicted by a dot. Each simulation run produced 100 cfDNA samples. The number of iterations of CelFiE was reduced to 100 iterations for computational efficiency. Simulated methylation ratios were unbiased.

***Robustness to the choice of markers***


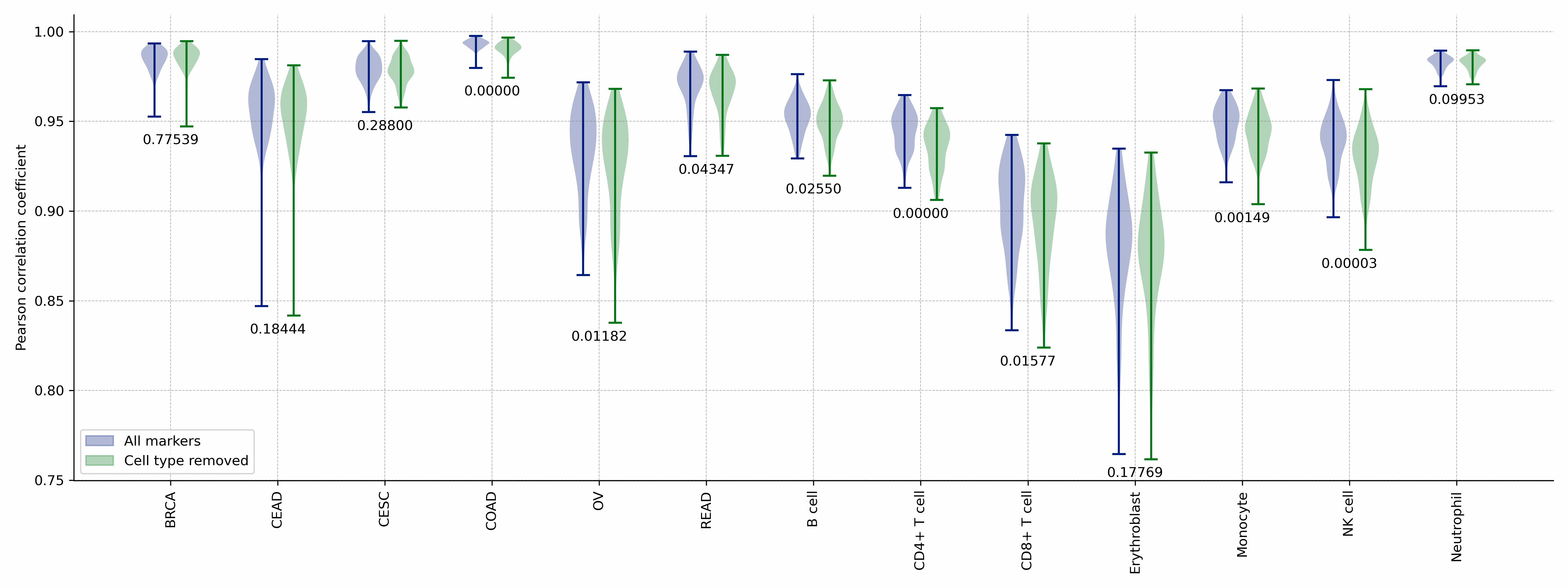
**Supplementary figure 7**: Distribution of Pearson correlation coefficients of each cell type based on NNLS, before and after removing the markers specific to this cell type. Distribution is based on 100 simulation repeats. Difference between the 2 settings has been assessed using a one-sided T test, and *p*-value has been reported for each cell type. Each simulation run produced 100 cfDNA samples.


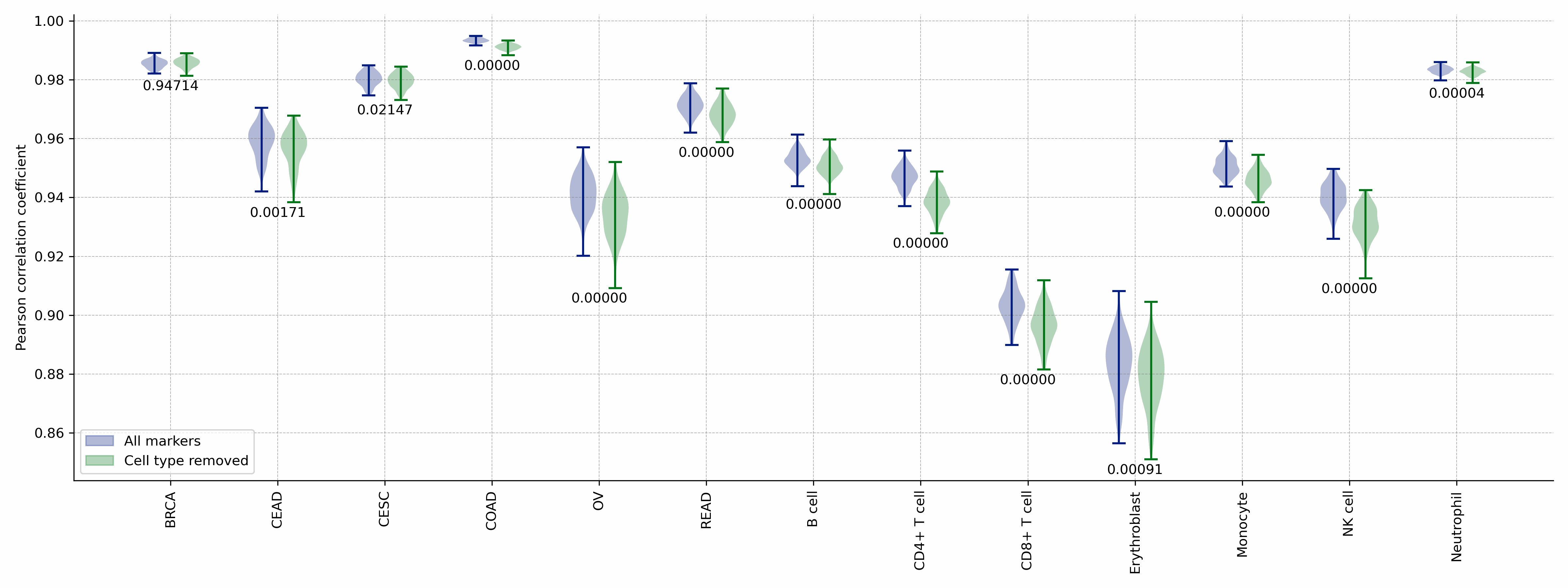
Supplementary figure 8: Distribution of Pearson correlation coefficients of each cell type based on NNLS, before and after removing the markers specific to this cell type. Distribution is based on 100 simulation repeats. Difference between the 2 settings has been assessed using a one-sided T test, and *p*-value has been reported for each cell type. Each simulation run produced 1000 cfDNA samples.
